# Integration of zinc anode and cement: unlocking scalable energy storage

**DOI:** 10.1093/nsr/nwae309

**Published:** 2024-09-04

**Authors:** Zhaolong Liu, Pan Feng, Ruidan Liu, Long Yuan, Xiangyu Meng, Guanghui Tao, Jian Chen, Qianping Ran, Jinxiang Hong, Jiaping Liu, Changwen Miao

**Affiliations:** Jiangsu Key Laboratory of Construction Materials, School of Materials Science and Engineering, Southeast University, Nanjing 211189, China; Jiangsu Key Laboratory of Construction Materials, School of Materials Science and Engineering, Southeast University, Nanjing 211189, China; Jiangsu Key Laboratory of Construction Materials, School of Materials Science and Engineering, Southeast University, Nanjing 211189, China; Jiangsu Key Laboratory of Construction Materials, School of Materials Science and Engineering, Southeast University, Nanjing 211189, China; Jiangsu Key Laboratory of Construction Materials, School of Materials Science and Engineering, Southeast University, Nanjing 211189, China; Jiangsu Key Laboratory of Construction Materials, School of Materials Science and Engineering, Southeast University, Nanjing 211189, China; Jiangsu Key Laboratory of Construction Materials, School of Materials Science and Engineering, Southeast University, Nanjing 211189, China; Jiangsu Key Laboratory of Construction Materials, School of Materials Science and Engineering, Southeast University, Nanjing 211189, China; State Key Laboratory of High Performance Civil Engineering Materials, Nanjing 210008, China; State Key Laboratory of High Performance Civil Engineering Materials, Nanjing 210008, China; Jiangsu Sobute New Materials Co., Ltd., Nanjing 211103, China; Jiangsu Key Laboratory of Construction Materials, School of Materials Science and Engineering, Southeast University, Nanjing 211189, China; State Key Laboratory of High Performance Civil Engineering Materials, Nanjing 210008, China; Jiangsu Key Laboratory of Construction Materials, School of Materials Science and Engineering, Southeast University, Nanjing 211189, China; State Key Laboratory of High Performance Civil Engineering Materials, Nanjing 210008, China

**Keywords:** structural energy storage, aerated cement mortar, zinc-ion hybrid supercapacitor

## Abstract

The significant volume of existing buildings and ongoing annual construction of infrastructure underscore the vast potential for integrating large-scale energy-storage solutions into these structures. Herein, we propose an innovative approach for developing structural and scalable energy-storage systems by integrating safe and cost-effective zinc-ion hybrid supercapacitors into cement mortar, which is the predominant material used for structural purposes. By performing air entrainment and leveraging the adverse reaction of the ZnSO_4_ electrolyte, we can engineer an aerated cement mortar with a multiscale pore structure that exhibits dual functionality: effective ion conductivity in the form of a cell separator and a robust load-bearing capacity that contributes to structural integrity. Consequently, a hybrid supercapacitor building block consisting of a tailored cement mortar, zinc metal anode and active carbon cathode demonstrates exceptional specific energy density (71.4 Wh kg^−1^ at 68.7 W kg^−1^), high areal energy density (2.0 Wh m^−2^ at 1.9 W m^−2^), favorable cycling stability (∼92% capacity retention after 1000 cycles) and exceptional safety (endurance in a 1-hour combustion test). By demonstrating the scalability of the structural energy-storage system coupled with solar energy generation, this new device exhibits great potential to revolutionize energy-storage systems.

## Teaser

We innovatively combined cement, the world's most ubiquitous construction material, with safe zinc electrodes to pioneer a structural energy storage system, unlocking scalable energy storage possibilities from civilian facilities to large-scale infrastructure.

## INTRODUCTION

**Figure 1.**
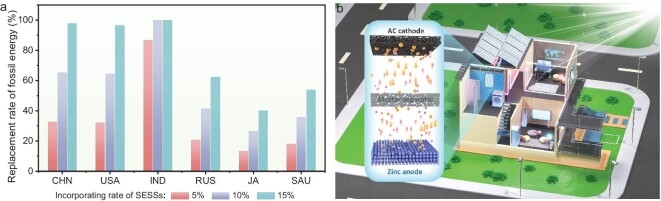
(a) Assessing the potential impact of integrating structural energy-storage systems (SESSs) into 10% of residential constructions on fossil electricity replacement rates across major countries and regions worldwide. (b) Conceptual schematic of a SESS utilizing ZIHCs: solar energy storage in a ZIHC-based wall, powering purple-highlighted electrical appliances in a net-zero energy building.

The rapid development of renewable energy sources, such as wind, solar and tidal wave sources, has expedited the substitution of fossil fuels and revealed vast opportunities for application [1,2]. However, the mismatch between energy generation and demand underscores the need for large-scale, secure and cost-effective energy-storage solutions to optimize the utilization of renewable energy [3]. Unfortunately, existing energy-storage methods, such as lithium-based power stations and pumped hydro storage, face significant constraints primarily due to the cost and safety of the systems or geographic limitations in the application area [4,5]. Cement-based materials, including cement paste, mortar and concrete, are the most widely used materials in structures worldwide, featured by low cost, abundant sources and outstanding durability [6,7]. Therefore, the integration of energy-storage components into cement-based structures to create efficient structural energy-storage systems (SESSs) can alleviate the occupation of limited space, reduce the complexity of designing highly intensive conventional electrochemical energy-storage systems and create new possibilities across a variety of applications, ranging from civilian facilities to large-scale infrastructure. This integration has the potential to redefine and revolutionize the environment that sustains our daily lives. For instance, by incorporating SESSs with an average energy density of 0.2 kWh m^−3^ into just 10% of residential constructions, significant reductions in reliance on fossil electricity could be achieved across major countries and regions worldwide. As illustrated in Fig. 1a, the potential for reducing fossil electricity consumption is substantial, with replacement rates of 65.3%, 64.4% and 100% for the top three consumers, namely China, the USA and India, respectively.

Batteries and supercapacitors are two popular energy-storage systems characterized by their distinct charging mechanisms and performance attributes [8]. For instance, supercapacitors are known for their high power density, extended cycling life and low energy density, while batteries exhibit the opposite characteristics [9,10]. Currently, cement-based materials are commonly deployed as separators and load supporters in supercapacitor configurations that have a ‘sandwich’ structure, with electrodes connected on both sides. Despite the utilization of pseudo-capacitor materials, such as MnO*_x_*, NiO*_x_* and FeO*_x_* [11–14], their insufficient energy densities and relatively high costs continue to hinder their widespread application on large scales and in different environments [15–18].

Zinc-ion hybrid capacitors (ZIHCs) represent relatively new systems that combine the Faradaic reaction of zinc anodes with the non-Faradaic adsorption/desorption of cathode capacitors (e.g. active carbon (AC)) to enhance the energy density, power density and lifespan [19]. The utilization of metallic zinc not only boosts the energy density due to its favorable electrochemical characteristics (gravimetric capacity of 823 mAh g^−1^, volumetric capacity of 5845 Ah L^−1^ and redox potential of −0.76 V vs. the standard hydrogen electrode) [20–22], but also enables compatibility with aqueous electrolytes that can be incorporated into cement-based systems. Moreover, the low cost and abundant availability of zinc, aqueous electrolytes, AC and cement, coupled with their eco-friendliness and safety, make the ZIHC a highly competitive candidate for commercialization in large-scale electrochemical energy-storage applications [21,23,24].

To rationally design a ZIHC-based SESS, the cement used must meet electrochemical and structural requirements; that is, it must be able to serve as both a mechanical support and a separator. As a mechanical support, it is essential for the cement to reduce the porosity and number of defects to maintain a high strength. In its role as a separator, it is favorable for cement to have a high wettability and good liquid penetration ability to enhance the ionic conductivity of the electrolyte. Typically, cement-based materials contain numerous micropores and nanopores, with a volume fraction ranging from 10% to 30% [25]. However, most of these pores are not interconnected, resulting in a poor ion migration rate [26]. In fact, improving the ionic conductivity often comes at the expense of improving the mechanical properties, thus limiting the reliability of these materials as load-bearing structural components [27,28]. Therefore, the deliberate design of the pore structure within cement is crucial for the realization of high-performance SESSs.

Air entrainment is a convenient method for adjusting the pore structures of cementitious materials because it significantly improves the porosity with minimal interference in cement hydration. Surfactants, such as sodium dodecyl sulfate (SDS), cocamidopropyl betaine (CAB) and triterpenoid saponins (TTS), are used in physical methods to generate air bubbles during mixing. Surfactants create bubbles by reducing the interfacial energy and surface tension of a cement paste. Gas-generating agents, such as aluminum powder (AP), zinc powder and hydrogen peroxide (H_2_O_2_), are employed in chemical methods to produce gas through reactions with alkaline species in fresh pastes [27,29]. As mentioned above, the introduction of a large number of air pores often severely degrades the mechanical properties. Considering the alkaline hydration products in cementitious materials and the weak acid electrolyte utilized in ZIHCs, pore refinement may occur between them to compensate for strength, thus facilitating the development of aerated cementitious separators with enhanced mechanical properties and high ionic conductivities.

Herein, a novel structural energy-storage device is designed by ingeniously combining ZIHCs with an aerated mortar separator (AMS) that is vacuum-impregnated with various Zn^2+^-containing electrolytes (ZnSO_4_, ZnCl_2_, Zn(NO_3_)_2_, Zn(OAC)_2_, Zn(CF_3_SO_3_)_2_). The optimized aerated mortar using the ZnSO_4_ electrolyte exhibits an improved compressive strength (∼15 MPa) and high ionic conductivity (12.6 mS cm^−1^) due to the optimized multiscale pore structure of the mortar and adverse reaction of the electrolyte. Consequently, the assembled ZIHC device exhibits a very high specific energy density (71.4 Wh kg^−1^ at 68.7 W kg^−1^, calculated by AC mass), a high areal energy density (2.0 Wh m^−2^ at 1.9 W m^−2^) and good cycling stability (∼92% capacity retention after 1000 cycles). These outcomes illustrate a harmonious balance between electrochemical properties, mechanical properties and construction costs, indicating the promising potential of this strategy for the achievement of large-scale energy storage through infrastructure integration (Fig. 1b).

## RESULTS AND DISCUSSION

### Preparation and characterization of AMS

**Figure 2.**
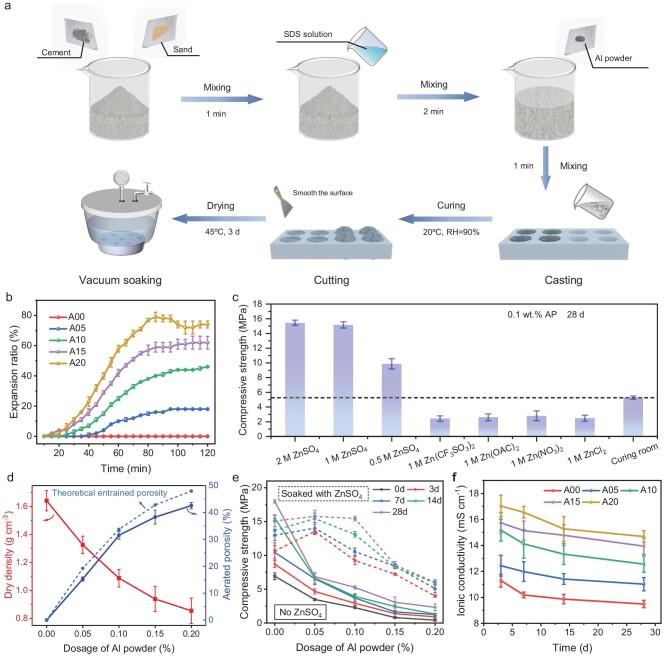
Preparation and characterization of AMS. (a) Preparation process of AMS. (b) Expansion ratio versus hydration time under various AP dosages. (c) Dry densities and aerated porosities of AMSs with varied AP dosage. (d) Compressive strengths of groups with 0.1 wt% AP after 28 days with various electrolytes. (e) Compressive strength varied with AP dosages under different curing time and conditions. (f) Ionic conductivity varied with soaking time in 2 M ZnSO_4_ among different groups.

A schematic presentation of the aerated mortar production process using physical air-entrainer (SDS) and chemical air-entrainer (AP) is depicted in Fig. 2a. The electrolyte solution was vacuum-impregnated into the AMS as the medium for ion migration under the applied electric field. The concentration of SDS at 0.015 wt% of cement is adopted to ensure optimal working efficiency [27,29,30]. To investigate the effects of AP, specimens with different AP dosage ratios (0, 0.05, 0.10, 0.15 and 0.20 wt% of cement) and a constant SDS dosage ratio (0.0015 wt%) were prepared and labeled as A00, A05, A10, A15 and A20, respectively, as listed in Table S1. The specimen ages were designated based on the time after drying.

Through the calculations of expansion rates (see Supplementary information Sections 1.7 and 1.8 for more details) [29], it was determined that SDS entrains approximately 3∼5 vol% air during initial mixing. The addition of AP significantly amplifies the expansion ratio (Fig. 2b) while simultaneously reducing the dry densities (Fig. 2c) due to increased gas generation. Notably, with an AP dosage of 0.1 wt%, the dry density of aerated mortar (A10) reaches 1.09 g cm^−3^, which accounts for nearly 33 vol% porosity compared with the A00 group. Furthermore, at dosages surpassing 0.1 wt%, there is a decline in air entraining efficiency due to bubble collapse at such high porosity [31].

To facilitate Zn^2+^ transport for ZIHC, the pore solution in aerated mortar needs be replaced by Zn^2+^-containing electrolytes. Among different salts tested, including ZnSO_4_, Zn(NO_3_)_2_, ZnCl_2_, Zn(OAC)_2_ and Zn(CF_3_SO_3_)_2_, the group soaked with ZnSO_4_, as shown in Fig. 2d, shows significantly higher compressive strength compared with the other groups. In addition, considering the electrochemical performance of these electrolytes in coin batteries (Fig. S1), a 2 M ZnSO_4_ electrolyte is deemed to be suitable for the establishment of structural ZIHCs devices.

To gain further insights into the effects of a 2 M ZnSO_4_ electrolyte on the mechanical properties of aerated mortar, the compressive strengths of groups with different AP dosages after varying soaking times are compared with those of groups without electrolyte soaking after different curing times. As shown in Fig. 2e, the group without electrolyte soaking exhibits a notable decrease in compressive strength with increasing AP dosage. At an AP dosage of 0.2 wt%, the 28d compressive strength is as low as 2.3 MPa, showing an 87% decline compared with that of the group without AP, falling short of load-bearing requirements. Conversely, in specimens soaked in ZnSO_4_ solution, a distinctive trend emerges in which the compressive strength initially rises and subsequently decreases with increasing AP dosage. Surprisingly, the compressive strength of the aerated mortar with ZnSO_4_ soaking exceeds that of specimens without ZnSO_4_ treatment by a significant margin. Furthermore, for AP dosages of <0.1 wt% and subjected to 28 days of ZnSO_4_ soaking, their compressive strengths approach those without added AP, reaching a stable value of ∼15 MPa. This phenomenon can be attributed to the gradual formation of connected pore structures with increasing AP dosage, which promotes the complete intrusion of ZnSO_4_ electrolyte into the pore structure, generating more products to enhance strength (Fig. S2). This suggests favorable mechanical properties for load-bearing applications, previously unreported, warranting further investigation into strengthening mechanisms. On the other hand, regarding specimens in the A00 group soaked in ZnSO_4_, their compressive strength surpasses those cured in a standard curing room during the initial 14 days. This can be attributed to the increased hydration degree resulting from sufficient and fast water supply under vacuum conditions [26]. As expected, specimens in the A00 group with 28 days of soaking show lower compressive strength than those without soaking, closely associated with surface cracking caused by the formation of expansive products under sulfate attack conditions [32].

Figure 2f illustrates the ionic conductivity of the aerated mortar soaked with 2 M ZnSO_4_ across different groups. The ionic conductivity rises from 9.5 to 14.7 mS cm^−1^ after a 28-day soaking, correlating with an increase in AP dosage from 0 to 0.2 wt%, indicating an improvement in pore structure facilitated by air entrainment. Meanwhile, the ionic conductivity gradually decreases over time and stabilizes at a steady-state value. For example, the A10 group records an ionic conductivity of 12.6 mS cm^−1^ after a 28-day soaking, marking a 17.3% decline compared with groups with a 3-day soaking (15.2 mS cm^−1^). This phenomenon can be attributed to pore refinement resulting from cement hydration and interactions between hydration products and electrolytes [26,33].

### Strengthening mechanism of mechanical properties and ionic conductivity of AMS

**Figure 3.**
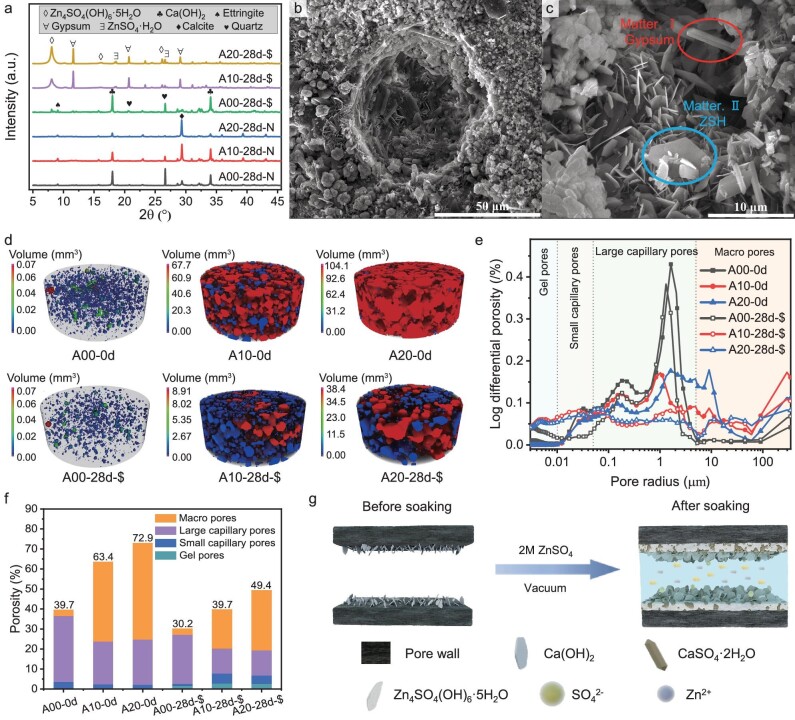
Characterization of AMS. (a) XRD patterns of various groups. (b) and (c) Scanning electron microscopy (SEM) images of reaction products for ‘A10-28d-$’ group at different magnifications. (d) 3D rendering of pore structure for various groups via X-CT. (e) Pore-size distribution of various groups via MIP method. (f) Total porosity and distribution of various groups. (g) Schematic diagram of the reaction mechanism between hydration products and ZnSO_4_.

The X-ray diffraction (XRD) results are presented in Fig. 3a and Fig. S3. The specimens in different groups without ZnSO_4_ soaking are labeled as ‘-N’, while their corresponding soaked groups are denoted as ‘-$’. XRD patterns of the ‘-N’ group reveal the presence of calcium hydroxide (CH) and calcite, resulting from cement hydration and carbonation due to the reaction of CH and CO_2_ in air [34], respectively. The peaks of quartz stem from the raw material sand. Following the second curing period of 28 days, the A00 group exhibits more pronounced CH peaks because of the ongoing hydration, whereas the intensity of the CH peaks decreases alongside heightened calcite peaks in the AP-containing groups. This phenomenon becomes stronger with increasing AP dosage, which introduces abundant pores and thus facilitates carbonation. Interestingly, in specimens soaked in 2 M ZnSO_4_ for 28 days, the AP-containing groups display the absence of CH, with new and distinct peaks of Zn_4_SO_4_(OH)_6_·5H_2_O (ZSH), ZnSO_4_·H_2_O and gypsum emerging. Given the disappearance of CH and the alkaline environment of the pore solution, ZSH and gypsum are suggested to be formed through the reaction between ZnSO_4_ and CH [33,35], while ZnSO_4_·H_2_O may have precipitated during the drying process. The formation of ZSH and gypsum is also corroborated by SEM observations (Fig. 3b and c) and energy dispersive spectrometer analysis (Fig. S4), in the former of which plenty of hexagonal and rod-shaped crystals are found in the aerated pores, providing strong evidence for the improved mechanical properties. Notably, the ‘A00-28d-$’ group exhibits a phase composition that is similar to that of the ‘A00-28d-N’ group. This can be attributed to the limited intrusion depth of ZnSO_4_ in the absence of AP, as demonstrated in Fig. S5, and the formed ZSH prevents further interaction with internal hydration products.

The pore structure of AMSs plays a critical role in determining their mechanical properties and ionic conductivities, often with conflicting requirements. In order to further elucidate the mechanisms that enhance mechanical properties and ionic conductivity, the structural evolution of large pores (radius >50 μm) and small pores (radius ranging from 0.005 to 300 μm) of AMS was investigated by using X-ray computed tomography (X-CT) and mercury intrusion porosimetry (MIP), respectively. The pore structure rendered via X-CT is illustrated in Fig. 3d. Initially, specimens in ‘A00-0d’ consisted of isolated pores with a volume of <0.07 mm^3^ due to entrained bubbles during mixing. In contrast, the pore structure of ‘A10-0d’ comprised predominantly interconnected large pore spaces in addition to with some isolated small pores (<0.2 mm), which greatly enrich routes for ion migration and yield higher ionic conductivity. Moreover, an increase in AP dosage leads to an expansion of the volume occupied by interconnected pores. Following vacuum impregnation with ZnSO_4_ for 28 days, the pore structure of ‘A00-28d-$’ exhibits negligible change. On the contrary, in aerated mortar that is fully reacted with ZnSO_4_, there is the apparent filling of macropores in addition to a reduction in large interconnected pores. Similar phenomena can be found in MIP results, in which detected pores are categorized into gel pores (0.005–0.01 μm), small capillary pores (0.01–0.05 μm), large capillary pores (0.05–5 μm) and macro pores (>5 μm) [36]. As shown in Fig. 3e, in aerated mortar, macro pores in groups with ZnSO_4_ demonstrate a more significant decrease than those under standard curing conditions, which can be attributed to the pore-refining effect of ZSH formation. Meanwhile, compared with the initial state, the average size and volume of macro pores and capillary pores decrease, while the volume of gel pores increases [37], which may be caused by the cement hydration and indicates that the filling effect of ZnSO_4_ is only significant for macro pores (Fig. 3e and Fig. S11b). By combining the results of CT (>50 μm) and MIP (<50 μm), the total porosity and contributions of all kinds of pores are summarized in Fig. 3f. With continuous soaking in ZnSO_4_ solution, the volume of small capillary pores and gel pores increase while the porosity attributed to macro pores and large capillary pores decreases, leading to a marked reduction in total porosity. Consequently, it can be inferred that the mechanical properties, including fracture toughness, will also improve [38,39].

In summary, the comprehensive mechanism behind the enhancement of mechanical properties and ionic conductivity in aerated mortar with ZnSO_4_ electrolyte is illustrated in Fig. 3g. By incorporating AP, large and interconnected pores are generated in AMSs for ions migration. Initially, reactive hydration products such as CH are dispersed randomly in the microstructure. Upon vacuum impregnation with ZnSO_4_ electrolyte, the reaction between ZnSO_4_ and CH gives rise to ZSH and gypsum. Depending on the dosage of AP, pores of specific sizes are filled with these newly formed products, to some degree preventing the deeper penetration of reactive agents. Consequently, a new equilibrium is reached between the altered microstructure and the electrolyte, with a certain residual concentration of ZnSO_4_ solution remaining in the pore channels. This equilibrium results in AMSs with optimized mechanical properties and electrochemical properties. Moreover, the pore-refining effects, especially for macropores, greatly increase the mechanical properties of AMSs at an acceptable sacrifice of ionic conductivity.

### Electrochemical properties

Symmetrical batteries using zinc electrodes were initially assembled to assess the compatibility of the AMS. Fig. S12 illustrates the polarization voltage curves of Zn plating/stripping at a current density of 1.0 mA cm^−2^ with a capacity of 0.5 mAh cm^−2^. During the first 100 hours, all cells using AMS exhibit relatively high polarization compared with those using commercial separators (CS). Additionally, the polarization voltage decreases with an increase in AP dosage. The results reveal that, although the ionic conductivity of AMSs is high, their substantial thicknesses (∼5 mm) contribute to increased resistance and ohmic polarization [40,41]. In terms of long-term cycling stability, cement-based separators demonstrate extended cycling durations before short-circuit occurrence due to their elevated hardness and thickness that prevent dendrite growth [42,43].

**Figure 4.**
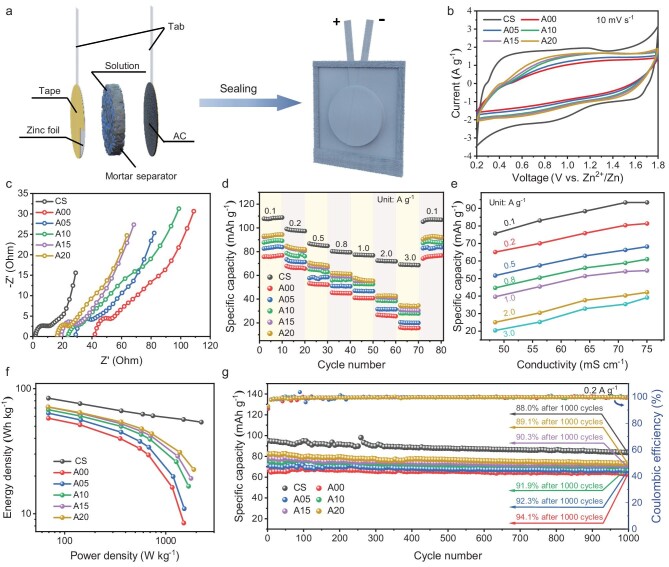
Electrochemical performance of ZIHCs structural energy-storage device. (a) Fabrication of the ZIHCs structural energy-storage device. (b) CV curves of structural energy-storage devices with various AP dosages at 10 mV s^−1^. (c) Nyquist profiles of ZIHCs coin battery with commercial separator and structural energy-storage devices with various AP dosages at OCV. (d) Rate performance of ZIHCs coin battery with commercial separator and structural energy-storage devices with various AP dosages. (e) Relationships between specific capacities and conductivities. (f) Energy densities versus power densities with various separators. (g) Long-term cycling performance of ZIHCs coin battery with commercial separator and structural energy-storage devices with various AP dosages measured at 0.2 A g^−1^.

As shown in Fig. 4a, the ZIHCs with AMS were further fabricated using a zinc foil anode and AC@SSM cathode and then well sealed in an Al-plastic package to maintain high electrolyte saturation and prevent matter exchange with the external environment. Their electrochemical performances were thoroughly characterized and compared with those of CR2032 coin cells employing CS. The CV curves of ZIHCs at 10 mV s^−1^ in Fig. 4b display similar shapes to those of coin cells, demonstrating stable workability within the voltage range of 0.2–1.8 V. With the increase in AP dosage, the area encompassed by the CV curve expands, indicating enhanced capacity. Upon the gradual escalation of scan rates to 100 mV s^−1^, the shapes of the CV curves transition from rectangle to fusiform due to the mismatch between the low electrochemical reaction rate and the high scan rate (Fig. S13a–c). The Nyquist plots of different groups at open-circuit voltage (OCV) by using electrochemical impedance spectroscopy tests, as depicted in Fig. 4c, exhibit a semicircle in the high-frequency region, a nearly horizontal line in the middle-frequency region and two connected diagonal lines with distinct gradients in the low-frequency region. With the increase in AP dosage, the solute resistance (*R*_s_) decreases from 40.98 to 16.54 Ω, which is higher than that observed when using CS (1.77 Ω), which is consistent with the CV results.

The rate performance of ZIHCs was investigated at different current densities from 0.1 to 3.0 A g^−1^ (Fig. 4d). When compared with the cell using CS, the groups with mortar separators show acceptable capacity loss at low current densities but experience a rapid decline at high current densities, which is mitigated by increasing the AP dosage. It is evident that higher AP dosage results in enhanced specific capacities, especially at elevated current densities. For example, the A00 group without AP shows an average specific capacity of 76.0 mAh g^−1^ (calculated by AC mass) at 0.1 A g^−1^ and 15.7 mAh g^−1^ at 3.0 A g^−1^. In contrast, the A20 group with a 0.2% AP dosage exhibits capacities of 93.8 mAh g^−1^ at 0.1 A g^−1^ and 34.2 mAh g^−1^ at 3.0 A g^−1^ under the same conditions, representing increases of 23.4% and 117.8%, respectively. The disparity may be attributed to the enhanced ionic conductivity of AMSs. As shown in Fig. 4e, the specific capacities of ZIHCs demonstrate an almost linear relationship with their ionic conductivities; similar slopes indicate comparable capacity losses caused by decreased conductivities. The internal resistance drops in galvanostatic charge–discharge (GCD) profiles significantly increase with rising current densities and decreasing AMS conductivities, leading to a decline in specific capacities (Fig. S14). Furthermore, the energy densities for ZIHCs employing AMSs are generally lower than those using CS and demonstrate a sharp decrease as power densities increase (Fig. 4f). However, the energy density at high power density can be improved through the adoption of AP dosages, as illustrated by the case of group A10, which exhibits an energy density of 71.4 Wh kg^−1^ at 68.7 W kg^−1^ and 16.6 Wh kg^−1^ at 1.7 kW kg^−1^, as well as excellent mechanical properties. In addition, as shown in Fig. S15, the voltage retention rate of the A10 group after 100 hours of setting was ∼70.5%, warranting a need for further research to improve this metric.

The cycling stability of structural energy-storage devices, as integral components of buildings, is crucial due to their limited replaceability compared with traditional energy-storage devices. The cycling stabilities of ZIHCs are measured by using the capacity retention rate at a current density of 0.2 A g^−1^ (Fig. 4g). After 1000 cycles, the capacity retention rates decrease with increasing AP dosage, with all remaining above 89.1%, surpassing that of devices using CS (at only 88.0%). The main reason for the capacity decline could be attributed to the formation of by-products on Zn foil, such as ZSH (Fig. S16). This difference in capacity retention can be attributed to the depth of discharging, which is reflected by specific capacities. Higher specific capacities result in a greater amount of reacted zinc on the anode and absorbed ions on the cathode, potentially leading to more pronounced growth of zinc dendrites on the anode and formation of irreversible ZSH on the cathode, consequently causing a faster decline in capacities. Furthermore, the coulombic efficiency remains nearly constant at ∼100%, particularly after ∼300 cycles, implying a high level of reversibility during the electrochemical process. Notably, over the course of ≤2000 cycles, groups utilizing mortar separators consistently demonstrate reliable performance without experiencing any short circuits. In contrast, the group using CS encounter short-circuiting after ∼1000 cycles, distinctly highlighting the superior short-circuit prevention capability of mortar separators (Fig. S17).

### Demonstration of larger ZIHC structure energy-storage systems

**Figure 5.**
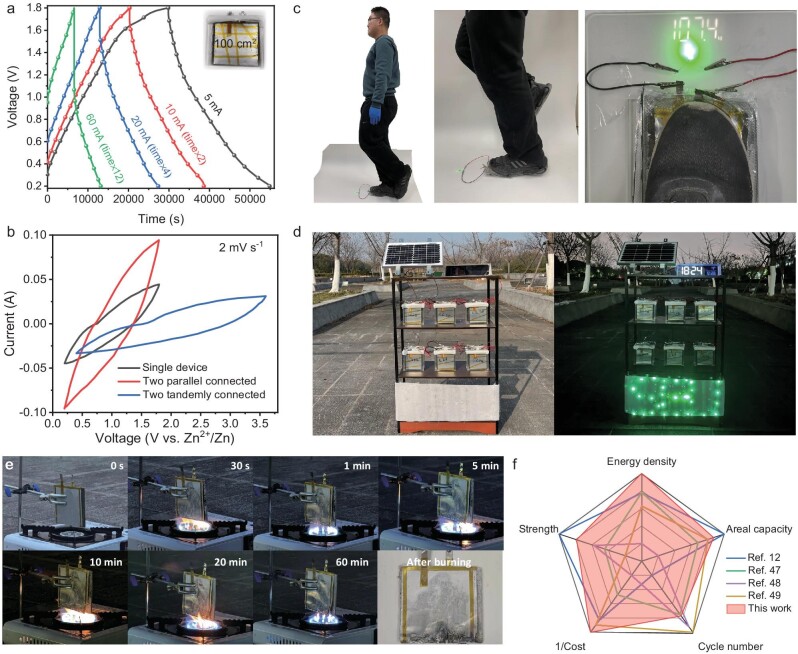
Electrochemical performance and demonstration of ZIHCs structural energy-storage device on a large scale. (a) Galvanostatic charge–discharge profiles for ZIHCs structural energy-storage device at currents ranging from 5 to 60 mA. (b) CV curves of a single 100-cm^2^ device, two parallel collected 100-cm^2^ devices and two tandemly connected 100-cm^2^ devices at 2 mV s^−1^. (c) Photograph of two tandemly connected 100-cm^2^ devices illuminating a green light-emitting diode with compressive load applied. (d) Photograph of a conceptual device for the integration of solar energy and SESS during day and night. (e) Combustion tests for a 100-cm^2^ unit of SESS. (f) Radar plots for performance comparison of this work and related references on the vital parameters of structural energy-storage devices.

Consequently, aerated mortar separators exhibit high mechanical properties and ionic conductivity, rendering them suitable for serving as both load carriers and separators in structural energy-storage devices. To further validate the potential of the widespread application of ZIHCs based on AMS, a larger device with an area of 100 cm^2^ was fabricated by following the same methodology and sealed in either plastic pouches or acrylic boxes (Fig. S18). The charge–discharge profiles of the device at different current densities are shown in Fig. 5a. The CV curves of a single 100-cm^2^ device, two devices connected in tandem and two devices connected in parallel are presented in Fig. 5b. It can be observed that the 100-cm^2^ device operates within in a voltage range of 0.2–1.8 V, exhibiting increased capacity when connected in parallel and achieving high output from 0.4 to 3.6 V when linked in tandem, thereby demonstrating its potential for practical application through straightforward configurations.

The electrochemical behaviors under load of the structural energy-storage device have been investigated. Two tandem-connected 100-cm^2^ devices are sealed in transparent plastic pouches and steadily illuminate a green light-emitting diode while supporting a person weighing >100 kg. This practical demonstration illustrates the feasibility and reliability of applying the ZIHC devices for load support in real-world buildings (Fig. 5c).

To visually demonstrate the energy-storage capabilities of our devices, three 100-cm^2^ units were sealed in acrylic boxes, connected in parallel to create an energy-storage module. Two such groups are then tandemly connected to power an LED screen with a minimum power output of 50 mW. As shown in Fig. S19, the LED screen is illuminated constantly for >1.5 hours, affirming the robust capacities of our large-scale SESSs. In Fig. 5d, a concept combination system between solar energy generation and SESS is presented. A solar photovoltaic panel with a maximum output of 10 W is employed to harvest energy during daylight hours. Twenty-four 100-cm^2^ devices sealed in acrylic boxes are connected in parallel or tandem configurations to store electricity generated by the solar photovoltaic panel and supply it to illuminate multiple LED dipoles arranged in an ‘SEU’ array (Fig. S20). This set-up is directly installed outdoors at Southeast University, Nanjing, China and exposed to natural environmental conditions from December 2023 to January 2024 (Fig. S21). Throughout the 1-month outdoor trial, our device displayed consistent and reliable performance despite the varying weather conditions of snow, rain and sunshine, with temperatures ranging between –2°C and 22°C.

The flame-retardancy of our SESS is illustrated in Fig. 5e. A 100-cm^2^ unit was directly exposed to butane fire for 1 hour, during which no discernible signs of combustion were found and the structural integrity of the unit was fully maintained. The hydration products of the mortar, with C–S–H as the primary phase, pose little risk of ignition during burning. Additionally, the decomposition of ZSH and gypsum absorbs heat, generating water vapor that contributes to further retardation of combustion [44,45]. The porous nature of the mortar separator facilitates the rapid release of water vapor, mitigating the risk of explosion that is often observed in more densely packed matrices [46].

The structural energy-storage device introduced in this study, based on the combination of ZIHCs and aerated mortar, is the first to introduce metallic zinc electrodes in cement-based settings that can work reliably in a neutral electrolyte environment. A comparison between this work and state-of-the-art works is outlined in Fig. 5f [12,47–49]. In contrast to existing structural energy-storage systems that are designed for building applications within an aqueous environment, this novel SESS has the highest specific energy density, relatively high areal energy density and outstanding capacity retention over extended cycling periods. Although there are two ‘less good’ parameters—strength and cycle number—SESSs can be acceptable for less critical parts of buildings given the current strength levels. More importantly, the service life of SESSs could be further improved by surface treatment of the electrodes and regulation of the electrolyte [50,51]. Overall, considering the abundance, low cost and scalable synthesis route, the proposed ZIHCs-based SESS holds promise for renewable energy technologies.

## CONCLUSION

In summary, a cement-based structural energy-storage device that initially integrates ZIHCs with aerated mortar is created by the combination of physical and chemical air-entrainers. Benefitting from a highly interconnected pore structure, the aerated mortars that are vacuum-impregnated with ZnSO_4_ electrolyte display simultaneously enhanced ionic conductivity and mechanical properties. The aerated mortar, optimized with an AP dosage of 0.1 wt%, show improved compressive strength (∼15 MPa), high ionic conductivity (12.6 mS cm^−1^) and effective internal short-circuit protection as separators and load carriers. The reinforcing mechanism of the adoption of the ZnSO_4_ electrolyte is attributed to the formation of ZSH and gypsum, which can fill in the macro pores and refine the pore structure. Meanwhile, the remaining pores are saturated by the electrolyte under the negative pressure, boosting the ionic conductivity. Consequently, the energy-storage device exhibits excellent specific energy density (71.4 Wh kg^−1^ at 68.7 W kg^−1^), high areal energy density (2.0 Wh m^−2^ at 1.9 W m^−2^) and robust cycling stability (∼92% capacity retention after 1000 cycles). Overall, the structural energy-storage devices show an optimized balance between electrochemical properties, mechanical properties and construction cost, demonstrating promising potential for large-scale energy storage. Considering the extensive inventory, buildings with electricity-storage capabilities could alleviate the limitations of renewable energy, thereby advancing progress toward a zero-carbon future.

## Supplementary Material

nwae309_Supplemental_File

## References

[1] Nastasi B, Markovska N, Puksec T et al. Techniques and technologies to board on the feasible renewable and sustainable energy systems. Renew Sust Energ Rev 2023; 182: 113428. 10.1016/j.rser.2023.113428

[2] Østergaard PA, Duic N, Noorollahi Y et al. Renewable energy for sustainable development. Renew Energy 2022; 199: 1145–52. 10.1016/j.renene.2022.09.065

[3] Chanut N, Stefaniuk D, Weaver JC et al. Carbon-cement supercapacitors as a scalable bulk energy storage solution. Proc Natl Acad Sci USA 2023; 120: e2304318120. 10.1073/pnas.2304318120 37523534 PMC10410735

[4] Trainer T . Some problems in storing renewable energy. Energy Policy 2017; 110: 386–93. 10.1016/j.enpol.2017.07.061

[5] Gür TM . Review of electrical energy storage technologies, materials and systems: challenges and prospects for large-scale grid storage. Energy Environ Sci 2018; 11: 2696–767. 10.1039/C8EE01419A

[6] Wang L, Tang S, Pia G et al. Editorial for special issue ‘fractal and fractional in cement-based materials’. Fractal Fract 2022; 6: 144–8. 10.3390/fractalfract6030144

[7] Mostafa AM, Yahia A. New approach to assess build-up of cement-based suspensions. Cem Concr Res 2016; 85: 174–82. 10.1016/j.cemconres.2016.03.005

[8] Lin Z, Goikolea E, Balducci A et al. Materials for supercapacitors: when Li-ion battery power is not enough. Mater Today 2018; 21: 419–36. 10.1016/j.mattod.2018.01.035

[9] Zhang L, Shi D, Liu T et al. Nickel-based materials for supercapacitors. Mater Today 2019; 25: 35–65. 10.1016/j.mattod.2018.11.002

[10] Chen T, Dai L. Carbon nanomaterials for high-performance supercapacitors. Mater Today 2013; 16: 272–80. 10.1016/j.mattod.2013.07.002

[11] Xu C, Zhang D. Multifunctional structural supercapacitor based on cement/PVA-KOH composite and graphene. J Compos Mater 2020; 55: 1359–69. 10.1177/0021998320969852

[12] Wang J, Zhan PM, Zhang D. Redox active cement-based electrolyte towards high-voltage asymmetric solid supercapacitor. Cem Concr Compos 2023; 138: 104987. 10.1016/j.cemconcomp.2023.104987

[13] Fang C, Zhang D. A large areal capacitance structural supercapacitor with a 3D rGO@MnO_2_ foam electrode and polyacrylic acid-Portland cement-KOH electrolyte. J Mater Chem A 2020; 8: 12586. 10.1039/D0TA03109G

[14] Fang C, Zhang D. Enhanced electrochemical matching between NiCo_2_O_4_/reduced graphene oxide and polymer cement electrolyte for structural supercapacitor. J Electrochem Soc 2022; 169: 060528. 10.1149/1945-7111/ac7752

[15] Gao Y, Yin J, Xu X et al. Pseudocapacitive storage in cathode materials of aqueous zinc ion batteries toward high power and energy density. J Mater Chem A 2022; 10: 9773. 10.1039/D2TA01014C

[16] Huang Y, Zeng Y, Yu M et al. Recent smart methods for achieving high-energy asymmetric supercapacitors. Small Methods 2017; 2: 1700230. 10.1002/smtd.201700230

[17] Wang Y, Song Y, Xia Y. Electrochemical capacitors: mechanism, materials, systems, characterization and applications. Chem Soc Rev 2016; 45: 5925. 10.1039/C5CS00580A 27545205

[18] Fang CQ, Zhang D. Electrochemical modulation of pore structure inside structural supercapacitor solid electrolyte. Microporous Mesoporous Mat 2022; 341: 112089. 10.1016/j.micromeso.2022.112089

[19] Dong L, Ma X, Li Y et al. Extremely safe, high-rate and ultralong-life zinc-ion hybrid supercapacitors. Energy Storage Mater 2018; 13: 96–102. 10.1016/j.ensm.2018.01.003

[20] Aristote NT, Deng X, Zou K et al. General overview of sodium, potassium, and zinc-ion capacitors. J Alloy Compd 2022; 913: 165216. 10.1016/j.jallcom.2022.165216

[21] Song M, Tan H, Chao D et al. Recent advances in Zn-ion batteries. Adv Funct Mater 2018; 28: 1802564. 10.1002/adfm.201802564

[22] Liu H, Wang JG, You Z et al. Rechargeable aqueous zinc-ion batteries: mechanism, design strategies and future perspectives. Mater Today 2021; 42: 73–98. 10.1016/j.mattod.2020.08.021

[23] Dong L, Yang W, Yang W et al. Multivalent metal ion hybrid capacitors: a review with a focus on zinc-ion hybrid capacitors. J Mater Chem A 2019; 7: 13810. 10.1039/C9TA02678A

[24] Song J, Xu K, Liu N et al. Crossroads in the renaissance of rechargeable aqueous zinc batteries. Mater Today 2021; 45: 191–212. 10.1016/j.mattod.2020.12.003

[25] He R, Ma H, Hafiz RB et al. Determining porosity and pore network connectivity of cement-based materials by a modified non-contact electrical resistivity measurement: experiment and theory. Mater Des 2018; 156: 82–92. 10.1016/j.matdes.2018.06.045

[26] Huang L, Tang L, Löfgren I et al. Moisture and ion transport properties in blended pastes and their relation to the refined pore structure. Cem Concr Res 2022; 161: 106949. 10.1016/j.cemconres.2022.106949

[27] Zhao RH, Tuan CY, Xu A et al. Conductivity of ionically-conductive mortar under repetitive electrical heating. Constr Build Mater 2018; 173: 730–9. 10.1016/j.conbuildmat.2018.04.074

[28] Zhao F, Hu J, Yang D et al. Study on the relationship between pore structure and uniaxial compressive strength of cemented paste backfill by using air-entraining agent. Adv Civ Eng 2021; 2021: 6694744. 10.1155/2021/6694744

[29] Tunstall LE, Ley MT, Scherer GW. Air entraining admixtures: mechanisms, evaluations, and interactions. Cem Concr Res 2021; 150: 106557. 10.1016/j.cemconres.2021.106557

[30] Silva BA, Ferreira Pinto AP, Gomes A et al. Suitability of different surfactants as air-entraining admixtures for lime mortars. Constr Build Mater 2020; 256: 118986. 10.1016/j.conbuildmat.2020.118986

[31] Du ZX, Zuo WQ, Wang PG et al. Ultralight, super thermal insulation, and fire-resistant cellular cement fabricated with Janus nanoparticle stabilized ultra-stable aqueous foam. Cem Concr Res 2022; 162: 106994. 10.1016/j.cemconres.2022.106994

[32] Wang Q, Wilson W, Scrivener K. Unidirectional penetration approach for characterizing sulfate attack mechanisms on cement mortars and pastes. Cem Concr Res 2023; 169: 107166. 10.1016/j.cemconres.2023.107166

[33] Mesecke K, Malorny W, Warr LN. Understanding the effect of sulfate ions on the hydrothermal curing of autoclaved aerated concrete. Cem Concr Res 2023; 164: 107044. 10.1016/j.cemconres.2022.107044

[34] Lei M, Deng S, Liu Z et al. Development of a sustainable CO_2_ solidified aerated concrete. ACS Sustain Chem Eng 2022; 10: 3990–4001. 10.1021/acssuschemeng.1c08695

[35] Chen H, Dai C, Xiao F et al. Reunderstanding the reaction mechanism of aqueous Zn-Mn batteries with sulfate electrolytes: role of the zinc sulfate hydroxide. Adv Mater 2022; 34: 2109092. 10.1002/adma.202109092 35137465

[36] Wang S, Zhang G, Wang Z et al. Effect of defoaming agent on the properties of cement mortars with hydroxyethyl methyl cellulose through adjusting air content gradient. Cem Concr Compos 2023; 139: 105024. 10.1016/j.cemconcomp.2023.105024

[37] Atahan HN, Oktar ON, Taşdemir MA. Effects of water-cement ratio and curing time on the critical pore width of hardened cement paste. Constr Build Mater 2009; 23: 1196–200. 10.1016/j.conbuildmat.2008.08.011

[38] Jang JG, Lee HK. Microstructural densification and CO_2_ uptake promoted by the carbonation curing of belite-rich Portland cement. Cem Concr Res 2016; 82: 50–7. 10.1016/j.cemconres.2016.01.001

[39] Suh H, Cho S, Her S et al. Influence of multi-scale three-dimensional pore characteristics on the mechanical properties of graphene oxide and carbon nanotube incorporated cement paste. Cem Concr Res 2023; 174: 107326. 10.1016/j.cemconres.2023.107326

[40] Zhu M, Ran Q, Huang H et al. Interface reversible electric field regulated by amphoteric charged protein-based coating toward high-rate and robust Zn anode. Nanomicro Lett 2022; 14: 219. 10.1007/s40820-022-00969-4 36355311 PMC9649586

[41] Shi H, Wang S, Fernandez C et al. Improved splice-electrochemical circuit polarization modeling and optimized dynamic functional multi-innovation least square parameter identification for lithium-ion batteries. Int J Energy Res 2021; 45: 15323. 10.1002/er.6807

[42] Garcia DCS, Wang K, Figueiredo RB. The influences of quartz content and water-to-binder ratio on the microstructure and hardness of autoclaved Portland cement pastes. Cem Concr Compos 2018; 91: 138–47. 10.1016/j.cemconcomp.2018.05.010

[43] Rafiz K, Murali DRL, Lin JYS. Suppressing lithium dendrite growth on lithium-ion/metal batteries by a tortuously porous γ-alumina separator. Electrochim Acta 2022; 421: 140478. 10.1016/j.electacta.2022.140478

[44] Xu YJ, Qu LY, Liu Y et al. An overview of alginates as flame-retardant materials: pyrolysis behaviors, flame retardancy, and applications. Carbohydr Polym 2021; 260: 117827. 10.1016/j.carbpol.2021.117827 33712167

[45] Morgan AB, Gilman JW. An overview of flame retardancy of polymeric materials: application, technology, and future directions. Fire Mater 2012; 37: 259–79. 10.1002/fam.2128

[46] Zhu Y, Hussein H, Kumar A et al. A review: material and structural properties of UHPC at elevated temperatures or fire conditions. Cem Concr Compos 2021; 123: 104212. 10.1016/j.cemconcomp.2021.104212

[47] Zhang EQ, Tang LP. Rechargeable concrete battery. Buildings 2021; 11: 103–17. 10.3390/buildings11030103

[48] Fang CQ, Zhang D. Pore forming with hemp fiber for magnesium phosphate structural supercapacitor. Mater Des 2020; 186: 108322. 10.1016/j.matdes.2019.108322

[49] Wang HM, Diao YF, Lu Y et al. Energy storing bricks for stationary PEDOT supercapacitors. Nat Commun 2020; 11: 3882. 10.1038/s41467-020-17708-1 32782258 PMC7419536

[50] Yin J, Zhang W, Alhebshi NA et al. Electrochemical zinc ion capacitors: fundamentals, materials, and systems. Adv Energy Mater 2021; 11: 2100201. 10.1002/aenm.202100201

[51] Wang Q, Zheng L, Ling Y et al. Thermo-electro dually activated carbon cloth as cathode material for aqueous hybrid zinc ion supercapacitor with ultrahigh stability and dramatically enhanced areal capacitance. Electrochim Acta 2023; 451: 142290. 10.1016/j.electacta.2023.142290

