# Supplementary material for: Integration of zinc anode and cement: unlocking scalable energy storage
Source: Natl Sci Rev. 2024 Sep 4;11(10):nwae309. doi: 10.1093/nsr/nwae309 (PMC11444079; doi:10.1093/nsr/nwae309)
Supplement: nwae309_Supplemental_File [file nwae309_supplemental_file.pdf]

Integration of Zinc Anode and Cement: Unlocking Scalable Energy Storage

Zhaolong Liu[1], Pan Feng[1]*, Ruidan Liu[1], Long Yuan[1], Xiangyu Meng[1], Guanghui Tao[1], Jian Chen[1]*, Qianping Ran[1,2], Jinxiang Hong[2,3], Jiaping Liu[1,2], Changwen Miao[1,2]*

（1. Jiangsu Key Laboratory of Construction Materials，School of Materials Science and Engineering，Southeast University，Nanjing 211189，China; 2. State Key Laboratory of High Performance Civil Engineering Materials, Nanjing 210008, China; 3. Jiangsu Sobute New Materials Co., Ltd., Nanjing 211103, China）

1 Supplementary materials and methods

1.1 Materials

Commercial activated carbon (AC, XFP01) with a specific area of 1800 m^2/g was purchased from XFNANO corporation. Polyvinylidene difluoride (PVDF) was purchased from AKEMA. Acetylene black was purchased from 3A. N-methylpyrrolidone (NMP), zinc chloride ($ZnCl_2$), zinc trifluoromethanesulfonate ($Zn(CF_3SO_3)_2$) and zinc acetate ($Zn(OAC)_2$) were purchased from Macklin. Zinc sulfate heptahydrate ($ZnSO_4 \cdot 7H_2O$) and sodium dodecyl sulfate (SDS) were purchased from Aladdin. Zinc nitrate ($Zn(NO_3)_2$) was purchased from XILONG SCIENTIFIC. All chemicals were of analytical grade and directly used without further purification.

Zn metal foil possessing a thickness of 50 μm and was purchased from Shanghai Weidi Metal Material Co., Ltd. Aluminum powder (AP) with reactive aluminum content at around 90 wt.% was purchased from LIGUANHUAGONG. The cement used in this research was reference cement (P. Ⅰ 42.5) purchased from Qufu Zhonglian Cement Co., Ltd. The sand used in this research was ISO standard sand purchased from Xiamen Aisiou Standard Sand Co., Ltd. Ultrapure water was used in all experiments.

1.2 Preparation of AC@SSM cathode

A slurry was prepared by mixing 80 wt% of AC, 10 wt% acetylene black and 10 wt% of PVDF in NMP under continuous stirring for more than two hours. The slurry was then drop-coated onto a stainless steel mesh (SSM, #304 with a mesh size of 74 μm) to form a film with a thickness of 20 μm. The coated mesh was dried in an oven at 60 ℃ for 12h, resulting in the AC electrode with an

average mass load of around 2.88 mg cm^{-2}.

1.3 Assembly of ZIHCs coin battery

The ZIHCs was assembled into CR2032 type coin cells for all tests in the open atmosphere. In this configuration, the AC electrode served as the working electrode, while Zn foil (50 μm in thickness, ~1.13 cm^2), commercial separator (glassy fiber membrane, Whatman Grade A), Zn-containing salt solution (e.g., 2M $ZnSO_4$) were adopted as the reference/counter electrode, separator, and electrolyte, respectively. The standard volume of electrolyte used was 150 μL, unless specified otherwise.

1.4 Electrochemical measurement

Cyclic voltammogram (CV) and electrochemical impedance spectroscopy (EIS) were performed on an electrochemical workstation (CHI 660C, HUACHEN) at room temperature (~20 ^oC). The voltage range for the CV test was set from 0.2 V to 1.8 V. The EIS test was conducted at open circuit potential with a voltage amplitude of 5 mV. Galvanostatic charging/discharging (GCD), rate, and cycling performance were measured by the battery cycler (CT3001A, LANHE). In this study, the constant current discharge mode was employed.

1.5 Calculation of electrochemical parameters

Specific capacity Q_S (mAh/g):

$$Q_S = \frac{1000 * I * t}{m}$$

where I, t, and m represents discharge current (A), discharge time (h), the mass (g) of active materials in the cathode, respectively.

Energy density E_S (Wh kg^{-1}):

$$E_S = \frac{1000 * \int IV(t)\, dt}{m}$$

where V(t), I, dt, and m represents discharge voltage (V), current (A), differential time (h), the mass (g) of active materials in the cathode, respectively.

Power density P_S (W kg^{-1}) :

$$P_S = \frac{E_S}{t}$$

where E_S and t represents energy density (Wh kg^{-1}) and discharge time (h), respectively.

The areal energy density E_A (Wh m^{-2}) and areal power density P_A (W m^{-2}) were calculated based on the following equations:

$$E_A = \frac{10000 * \int IV(t)\, dt}{A}$$

$$P_A = \frac{E_A}{t}$$

where V(t), I, dt, A, and t represents discharge voltage (V), current (A), differential time (h), the area (cm^2) of active materials in the cathode and discharge time (h), respectively. A=1.13 cm^2 in our electrodes.

1.6 Preparation of mortar separators

Firstly, a precise quantity of ultra-pure water was weighed, and SDS was dissolved in it by continuous mixing. At the same time, predetermined amounts of cement and sand were blended in a pot. Then, the mixture was mixed with the prepared SDS solution, stirred slowly for 30 s, followed by rapid stirring for 1.5 min. Subsequently, a calculated amount of AP was added to the obtained slurry and quickly stirred for an additional 1.5 min.

Next, a portion of the slurry was poured into cylindrical PTFE molds with a radius of 12 mm and a depth of 5 mm for electrochemical testing. Meanwhile, the rest of the slurry was cast into 20×20×20mm cubic stainless steel molds for specimens designated for mechanical property testing. All specimens with molds were placed in a curing room for 24h, with T=20℃ and RH=90%. Upon the completion of the curing process, a precision knife was employed to trim the excess mortar beyond the mold to attain a smooth surface.

Subsequently, the specimens were demolded and subjected to a 3-day drying in a vacuum drying oven at 45 ℃. Following the drying process, the specimens were vacuum impregnated with a Zn-containing salt solution (e.g., 2M $ZnSO_4$) within a vacuum tank. This marked the initiation of the second curing phase with a specified duration.

1.7 Measurement and calculation of expansion ratio

Upon the reaction of AP with alkaline substances dissolved from cement hydration, the generated hydrogen gas could inflate the slurry before its setting. Describing the influence of air

aeration, the expansion ratios of the slurry at specific time intervals were determined. In detail, volumetric cylinders with a maximum span of 10 mL were used for the test. Firstly, the slurry was cast into a volumetric cylinder with an initial volume of 5 mL and a cling film was used to seal the upper side. Observations were recorded at every 5-min interval. The expansion ratio at a certain time, denoted as $R_{\text{expan, t}}$ (%), was calculated by the formular,

$$R_{\text{expan,t}} = \frac{V_{\text{t}} - V_{\text{i}}}{V_{\text{i}}} \times 100$$

where V_t and V_i represents the volume (mL) at time t and at initial stage (mL), respectively. The experiment for each group was repeated for three times and the reported results were the average value of all tests for each group.

1.8 Measurement and calculation of dry density and aerated porosity

The dry densities for different groups were determined by weighing the 20×20×20 mm cubic specimens after the drying process. The dry density ρ_{dry} (g cm^{-3}) of a specimen can be calculated by the formular,

$$\rho_{\text{dry}} = \frac{m_{\text{cubic}}}{V_{\text{cubic}}}$$

where m_{cubic} and V_{cubic} represents the mass (g) and volume (cm^3) of a cubic specimen, respectively. In this study, V_{cubic} =8 cm^3. The experiment for each group was repeated for three times and the reported results were the average value of all tests for each group.

Following the measurement of expansion ratios t, the total volume increment was attributed to the aerated process. The aerated porosity p_{aerated} (%) can be calculated by the formular,

$$p_{\text{aerated}} = \frac{V_{\text{f}} - V_{\text{i}}}{V_{\text{f}}} \times 100$$

where V_{f} and V_{i} represent the final volume of the expanded slurry (mL) and initial volume (cm^3), respectively. The experiment for each group was repeated for three times and the reported results were the average value of all tests for each group.

According to the dry density of the group without AP, the theoretical aerated porosity p_{theo} of groups with AP can be calculated by the formular,

$$p_{\text{theo}} = \frac{\rho_0 - \rho'}{\rho_0}$$

where ρ_0 and ρ' represent the dry density of group without AP (g cm^{-3}) and dry density of a

specific group with AP (g cm^{-3}), respectively.

1.9 Measurement of compressive strength

The 20×20×20 mm cubic specimens were used for compressive strength measurement. The compressive strength was tested by UTM5105 microcomputer controlled electronic universal testing machine, with a loading speed of 0.5 mm min^{-1}. The maximum load was recorded to calculate the compressive strength *CS* (MPa) by the formular,

$$CS = \frac{P_{\max}}{A_{cub}}$$

where $P_{\max}$ and A_{cub} represent the maximum load applied in the test (N) and the surface area supporting the load (mm^2), respectively. In this study, A_{cub} =40 mm^2. The experiment for each group was repeated for three times and the reported results were the average value of all tests for each group.

1.10 Measurement of ionic conductivity

The cylinder mortar specimens were used for ionic conductivity measurement. Before testing, the mortar specimen served as separator was positioned between two pieces of stainless steel foil each with a thickness of 20 μm. The EIS tests were conducted on the assembled device under open-circuit voltage conditions. The results were recorded and used to calculate the ionic conductivity *IC* (mS cm^{-1}) by the formular,

$$IC = \frac{1000 d_{\mathrm{cy}}}{R_{\mathrm{s}} A_{\mathrm{ss}}}$$

where d_{cy}, R_{s}, and A_{ss} represent the thickness of the mortar separator (cm), solution resistance (Ω) and the surface area of the stainless steel foil (cm^2), respectively. In this study, d_{cy}=0.5 cm and A_{ss} =1.13 cm^2. The experiment for each group was repeated for three times and the reported results were the average value of all tests for each group.

1.11 Microscope characterization

The Bruker-AXS Discover X-ray diffractometer with the Cu target (wavelength: 1.5406 Å) was used for X-ray diffraction (XRD) analysis. The quantitative analysis of reaction and hydration products was performed by employing a STA 449F5 simultaneous thermal analyzer (STA) with a

temperature elevation speed of 10 ℃/min, and the elevated temperature ranged in 30-1000℃. The identification of phase variations during the temperature elevation was achieved by in-situ XRD measurement at 50, 200, 400, and 500℃ with the same temperature-varying procedure as in the STA measurement.

The morphology of the samples was observed using the Quanta 3DFEG field-emission scanning electron microscope (SEM) equipped with an EDX analyzer (Octane Elect Plus). The large-scale pores were visualized using the YXLON PRECISION S X-ray computed tomography (X-CT) while the small-scale pores of samples were detected by the Auto Pore IV 9520 mercury intrusion porosimetry (MIP).

1.12 Calculation of phases compositions

According to the results of STA and in-situ XRD analysis, the predominant reactions occurred in each stage are listed as follows:

Stage Ⅰ:

$$\mathrm{Ca(OH)_2 \rightarrow CaO + H_2O} \ \#(\mathrm{S1})$$

Stage Ⅱ:

$$\mathrm{CaCO_3 \rightarrow CaO + CO_2} \ \#(\mathrm{S2})$$

Stage Ⅲ:

$$\mathrm{Zn_4SO_4(OH)_6 \cdot 5H_2O \rightarrow ZnSO_4 \cdot 3ZnO \cdot 5H_2O + 3H_2O} \ \#(\mathrm{S3})$$

$$\mathrm{CaSO_4 \cdot 2H_2O \rightarrow CaSO_4 \cdot 0.5H_2O + 1.5H_2O} \ \#(\mathrm{S4})$$

Stage Ⅳ:

$$\mathrm{ZnSO_4 \cdot 3ZnO \cdot 5H_2O \rightarrow ZnSO_4 + 3ZnO + 5H_2O} \ \#(\mathrm{S5})$$

$$\mathrm{ZnSO_4 \cdot H_2O \rightarrow ZnSO_4 + H_2O} \ \#(\mathrm{S6})$$

Stage Ⅴ:

$$\mathrm{ZnSO_4 \rightarrow ZnO + SO_3} \ \#(\mathrm{S7})$$

As a result, the weight percentages of CH (w_{CH}, %) and $\mathrm{CaCO_3}$ (w_{CC}, %) in each group can be readily calculated by the following equations:

$$w_{\mathrm{CH}} = \frac{M_{\mathrm{CH}} \Delta m_1}{M_{\mathrm{H_2O}}}$$

$$w_{\mathrm{CC}} = \frac{M_{\mathrm{CC}} \Delta m_2}{M_{\mathrm{CO_2}}}$$

where Δm_1 and Δm_2 represent the mass loss in stage Ⅰ and stage Ⅱ, respectively. M_{CH}= 74.09, M_{CO2}=44.01, M_{H2O}=18.02, M_{CC}=100.09.

On the other hand, the weight relationships of $Zn_4SO_4(OH)_6 \cdot 5H_2O$ (w_{ZSH}, %), $CaSO_4 \cdot 2H_2O$ (w_{CS}, %) and $ZnSO_4 \cdot H_2O$ (w_{ZS}, %) in each group can be described by the following equations:

$$\frac{3w_{\mathrm{ZSH}}}{M_{\mathrm{ZSH}}}+\frac{3w_{\mathrm{CS}}}{2M_{\mathrm{CS}}}=\frac{\Delta m_3}{M_{\mathrm{H_2O}}}$$

$$\frac{5w_{\mathrm{ZSH}}}{M_{\mathrm{ZSH}}}+\frac{w_{\mathrm{ZS}}}{M_{\mathrm{ZS}}}=\frac{\Delta m_4}{M_{\mathrm{H_2O}}}$$

$$\frac{w_{\mathrm{ZSH}}}{M_{\mathrm{ZSH}}}+\frac{w_{\mathrm{ZS}}}{M_{\mathrm{ZS}}}=\frac{\Delta m_5}{M_{\mathrm{SO_3}}}$$

where Δm_3, Δm_4, and Δm_5 represent the mass loss in stage Ⅲ, stage Ⅳ and stage Ⅴ, respectively. M_{ZSH}= 551.24, M_{ZS}=179.45, M_{CS}=172.17, M_{SO3}=80.06.

The content of CH, including crystalline CH, carbonated CH, sulfated CH, serves as an indicator of the hydration degree. It was determined based on the measured weight of CH, $CaCO_3$, $CaSO_4 \cdot 2H_2O$ and the original sample weight, excluding any weight increase attributable to the introduction of $Zn_4SO_4(OH)_6 \cdot 5H_2O$, $ZnSO_4 \cdot H_2O$, additional $CaSO_4 \cdot 2H_2O$ and $CaCO_3$. The content of each CH type can be calculated by the following equations:

$$w_{\mathrm{intro}}=w_{\mathrm{CC}}\frac{M_{\mathrm{CO_2}}}{M_{\mathrm{CC}}}+w_{\mathrm{ZSH}}+w_{\mathrm{CS}}\frac{M_{\mathrm{CS}}-M_{\mathrm{CH}}}{M_{\mathrm{CS}}}+w_{\mathrm{ZS}}$$

$$w_{\mathrm{intri}}=100-w_{\mathrm{intro}}$$

$$C_{\mathrm{cry}}=\frac{w_{\mathrm{CH}}}{w_{\mathrm{intri}}}$$

$$C_{\mathrm{car}}=\frac{M_{\mathrm{CH}}w_{\mathrm{CC}}}{M_{\mathrm{CC}}w_{\mathrm{intri}}}$$

$$C_{\mathrm{sul}}=\frac{M_{\mathrm{CH}}w_{\mathrm{CS}}}{M_{\mathrm{CS}}w_{\mathrm{intri}}}$$

where w_{intro}, w_{intri}, C_{cry}, C_{car} and C_{sul} represent the weight introduced into the system (%), original weight of the system (%), crystalline CH content (%), carbonated CH content (%), sulfated CH content (%), respectively.

1.13 Fabrication of structural energy storage devices

Firstly, the zinc foil anode and AC@SSM cathode were individually connected to tabs using tape. Then, a mortar separator was placed between the two fabricated electrodes. Finally, the

connected electrodes and separator were sealed in an Al-plastic package by a vacuum heat-sealing machine.

1.14 Preparation of 100 cm^2 energy storage devices

An 11 cm × 11 cm × 0.5 cm aerated mortar separator with 0.1 wt.% AP was prepared using the same procedure mentioned above. The sizes of AC@SSM cathode and zinc foil anode were both 10×10 cm and were connected to tabs by tape. The aerated mortar separator was then inserted between the two electrodes and secured with tape. To clearly observe the working state of the structural battery, the connected electrodes and mortar separator were placed within a 12 cm ×12 cm × 0.8 cm acrylic box and sealed with tape and plastic films.

1.15 Evaluation of different properties for structural energy storage devices

To comprehensively assess the merits of our structural energy storage devices, we have selected normalized indicators with values ranging from 1 to 5. These indicators include energy density (F_{ED}), areal energy density (F_{AED}), cycle number (F_{CN}), reciprocal of cost (F_{RoC}), and compressive strength (F_{CS}). The calculation methods for each indicator are listed as follows,

$$F_{\mathrm{ED}} = 5 - \frac{4\left(E_{\mathrm{S,max}} - E_{\mathrm{S}}\right)}{E_{\mathrm{S,max}} - E_{\mathrm{S,min}}} \#(\mathrm{S8})$$

$$F_{\mathrm{AED}} = 5 - \frac{4\left(E_{\mathrm{A,max}} - E_{\mathrm{A}}\right)}{E_{\mathrm{A,max}} - E_{\mathrm{A,min}}} \#(\mathrm{S9})$$

$$F_{\mathrm{CN}} = 5 - \frac{4\left(N_{\mathrm{cy,max}} - N_{\mathrm{cy}}\right)}{N_{\mathrm{cy,max}} - N_{\mathrm{cy,min}}} \#(\mathrm{S10})$$

$$F_{\mathrm{RoC}} = 5 - \frac{4\left(Roc_{\mathrm{max}} - RoC\right)}{Roc_{\mathrm{max}} - Roc_{\mathrm{min}}} \#(\mathrm{S11})$$

$$F_{\mathrm{CS}} = 5 - \frac{4\left(CS_{\mathrm{max}} - CS\right)}{CS_{\mathrm{max}} - CS_{\mathrm{min}}} \#(\mathrm{S12})$$

where N_{cy} and RoC represent the cycle number when the capacity decreased by 90% and reciprocal of cost of structural energy storage devices for storing 1 Wh, respectively. The thorough results and detailed cost calculation are documented in Table S3-S4.

2 Supplementary figures

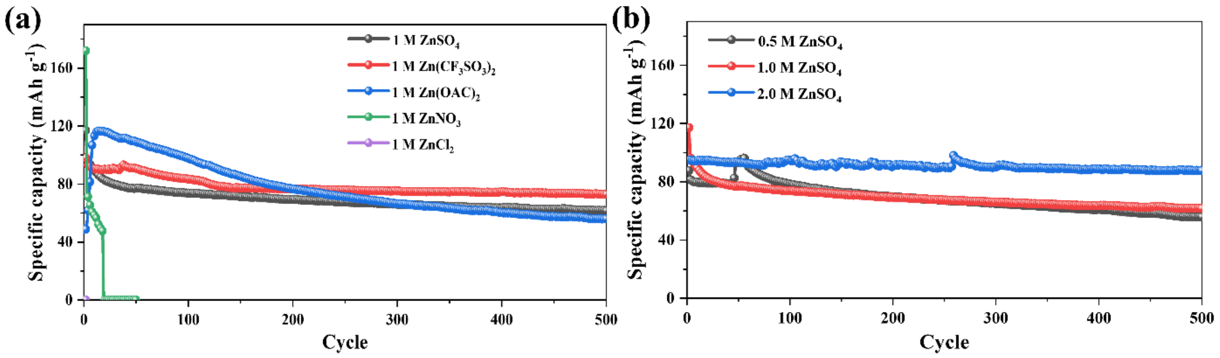

Fig. S1. Characterization of ZIHCs systems in various electrolyte. (a) Long-term cycling performance with various electrolytes. (b) Long-term cycling performance at different $ZnSO_4$ concentrations.

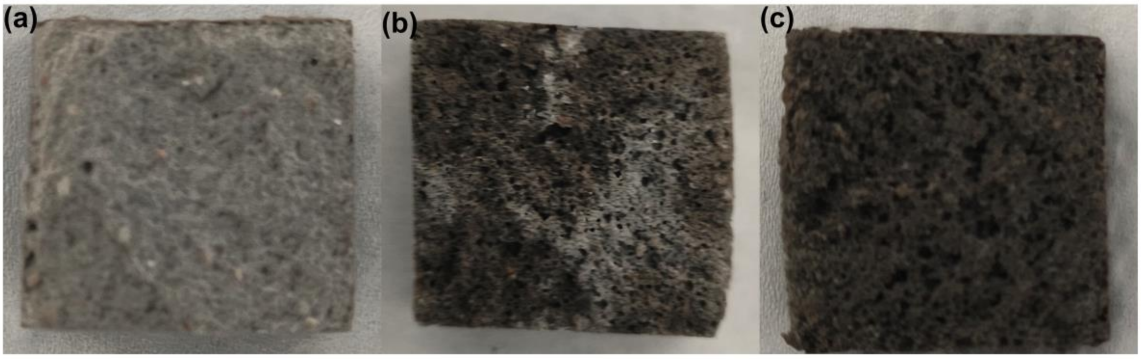

Fig. S2. Cross-sections of (a) A00, (b) A05, and (c) A10 groups after 28 days of soaking.

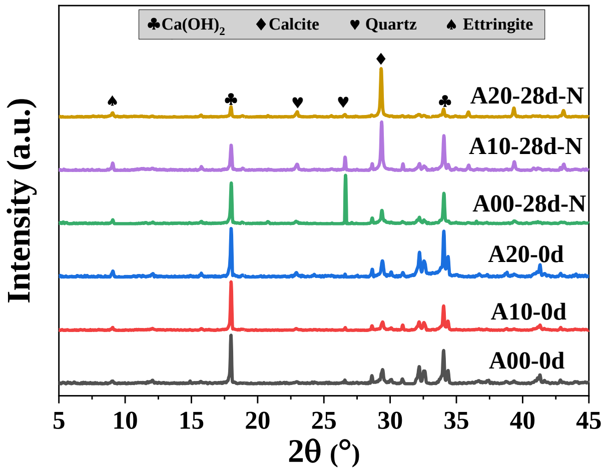

Fig. S3. XRD patterns of groups without $ZnSO_4$ soaking.

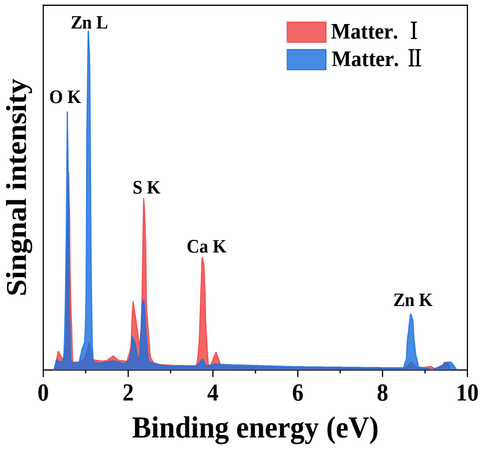

Fig. S4. EDS patterns of selected reaction products Matter. Ⅰ and Matter. Ⅱ

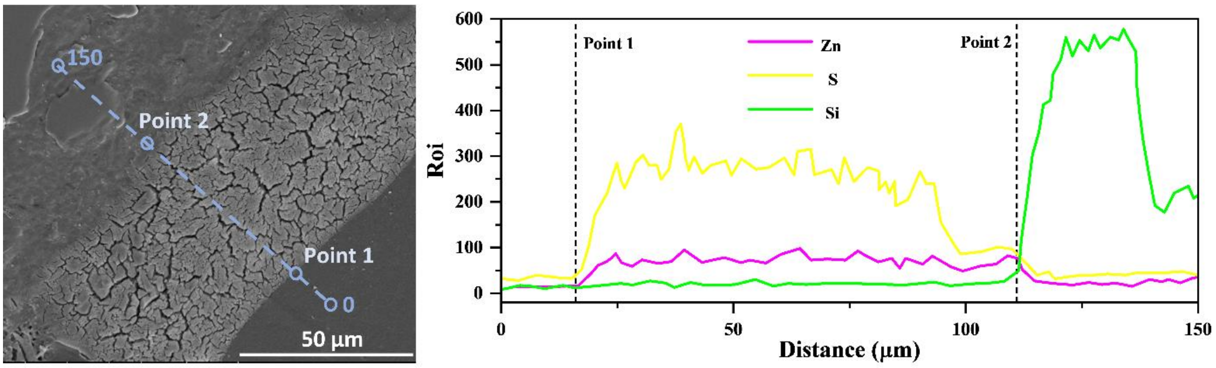

Fig. S5. Linear scan results of the "A00-28d-$" group on the outer surface.

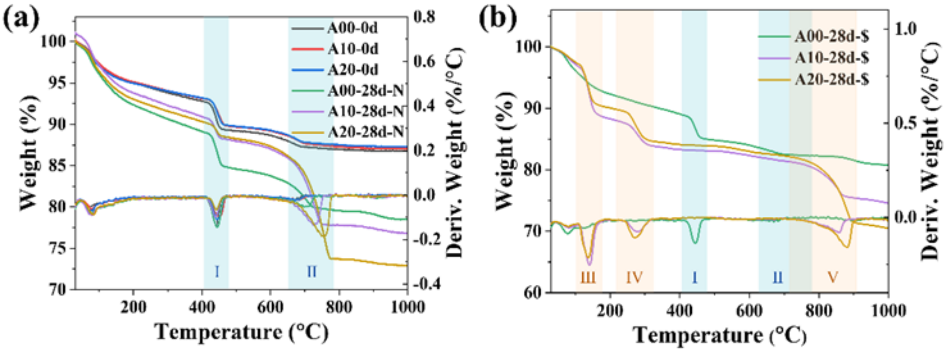

Fig. S6. TG and DTG curves for groups (a) without $ZnSO_4$ and (b) with $ZnSO_4$ soaking.

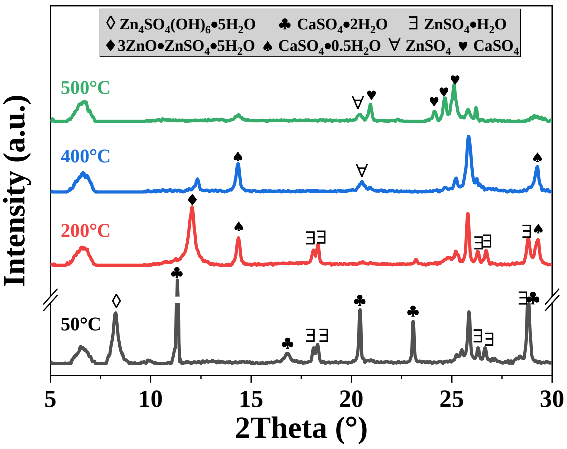

Fig. S7. In-situ XRD patterns of "A10-28d-\$" at elevated temperatures of 50, 200, 400, and 500℃.

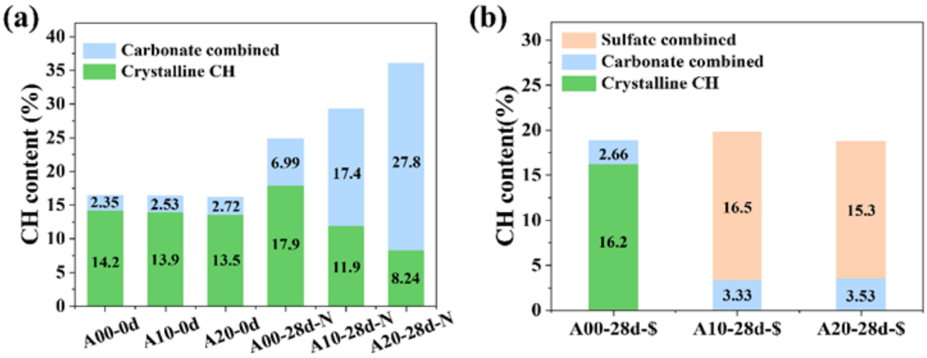

Fig. S8. Calculated CH contents for groups (a) without $ZnSO_4$ and (b) with $ZnSO_4$ soaking.

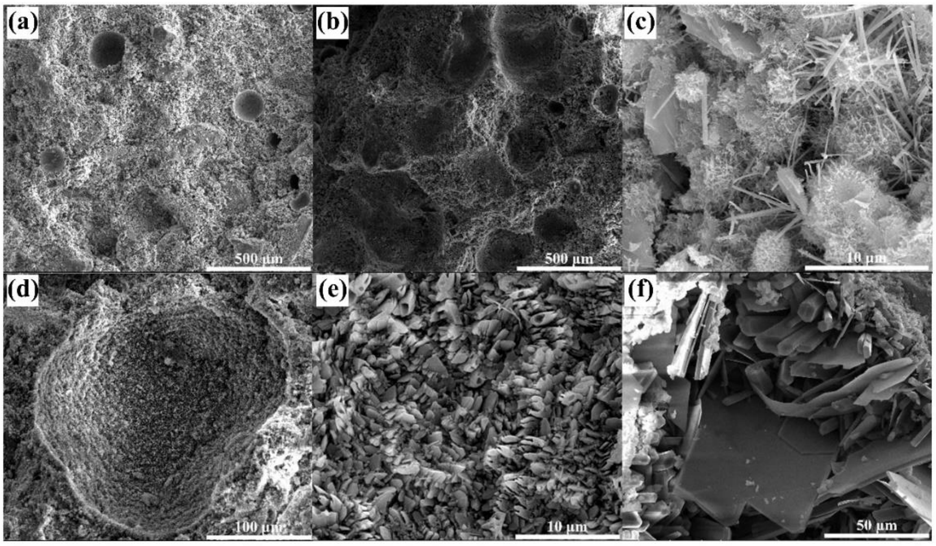

Fig. S9. SEM images of various groups. Macro pores distribution of (a) "A00-0d" and (b) "A20-0d". (c) Hydration products of "A00-28d-N". (d)-(e) Carbonation products of "A20-28d-N" at various magnifications. (f) Reaction products of "A20-28d-\$".

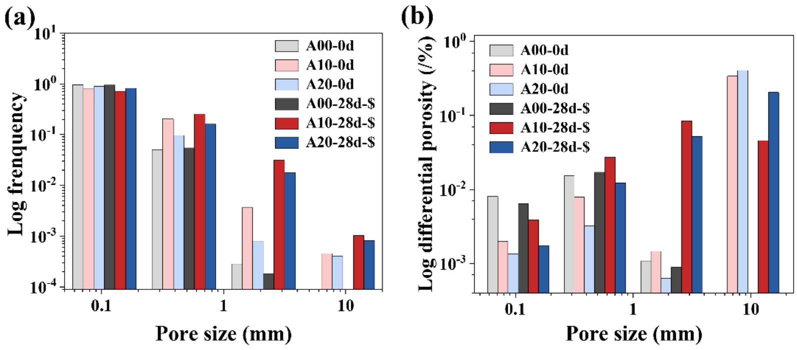

Fig. S10. Statistical analysis of pore size distribution of various groups via X-CT. (a) Frequency distribution of varied pore size. (b) Porosity distribution of varied pore size.

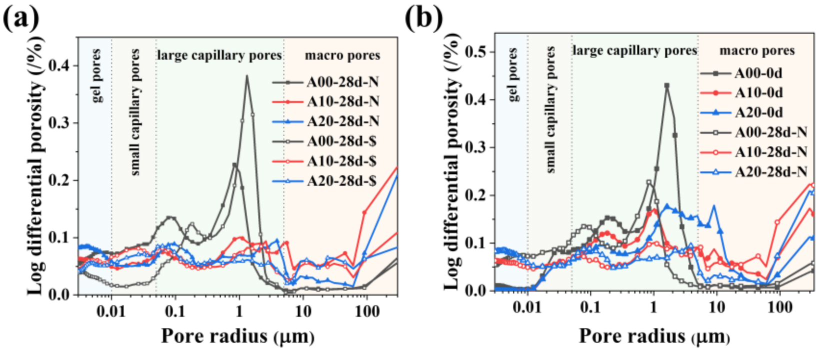

Fig. S11. Pore size distribution of various groups via MIP. (a) Comparation of groups with or without $ZnSO_4$ soaking at 28d. (b) Comparation of groups with different curing time.

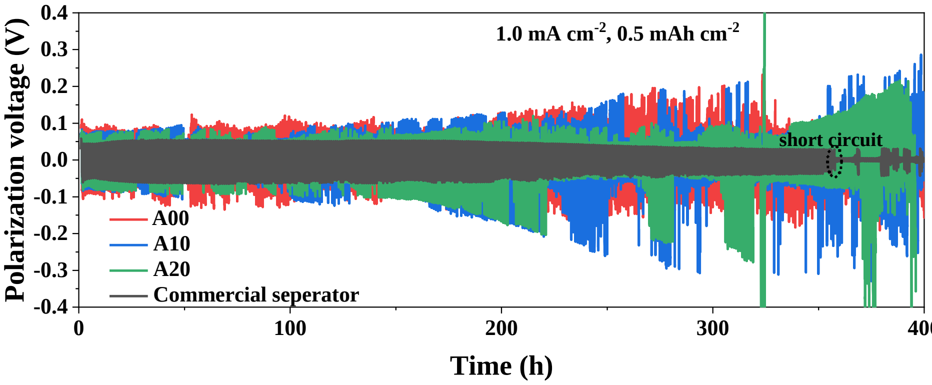

Fig. S12. Polarization voltage of the Zn plating/stripping at the current densities of 1.0 mA cm^{-2} with the capacities of 0.5 mAh cm^{-2}.

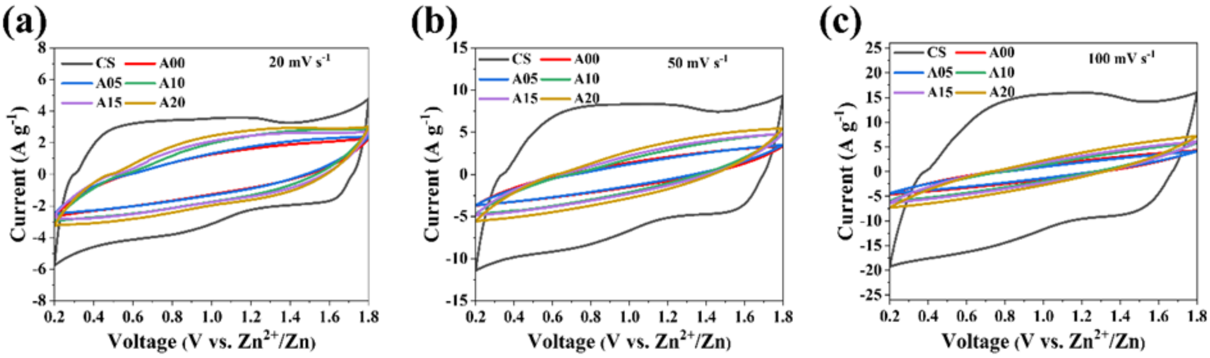

Fig. S13. CV curves of different groups with various separators at (a) 20 mV s^{-1}, (b) 50 mV s^{-1}, (c) 100 mV s^{-1}.

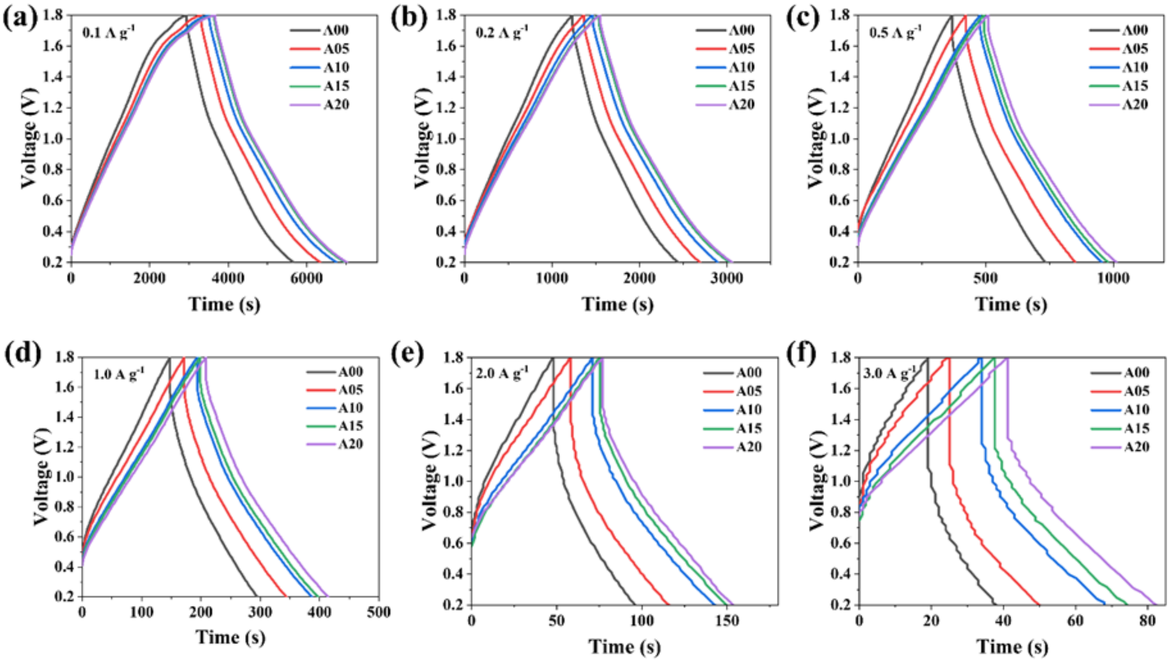

Fig. S14. GCD profiles of structural energy storage devices with various AP dosage at (a) 0.1 A g^{-1}, (b) 0.2 A g^{-1}, (c) 0.5 A g^{-1}, (d) 1.0 A g^{-1}, (e) 2.0 A g^{-1}, (f) 3.0 A g^{-1}.

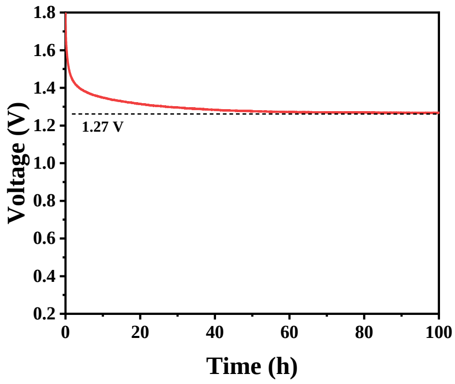

Fig. S15. Self-discharge curve of A10 group in 100 hours.

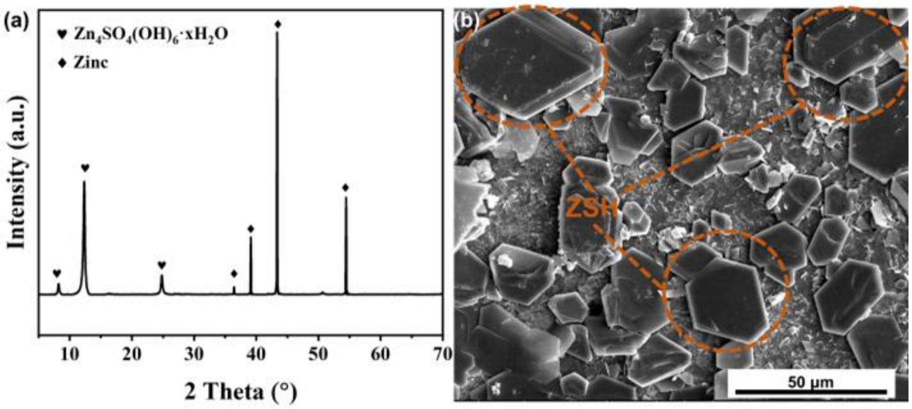

Fig. S16. Characterization of zinc anode after 1000 cycles, (a) XRD pattern and (b) SEM image.

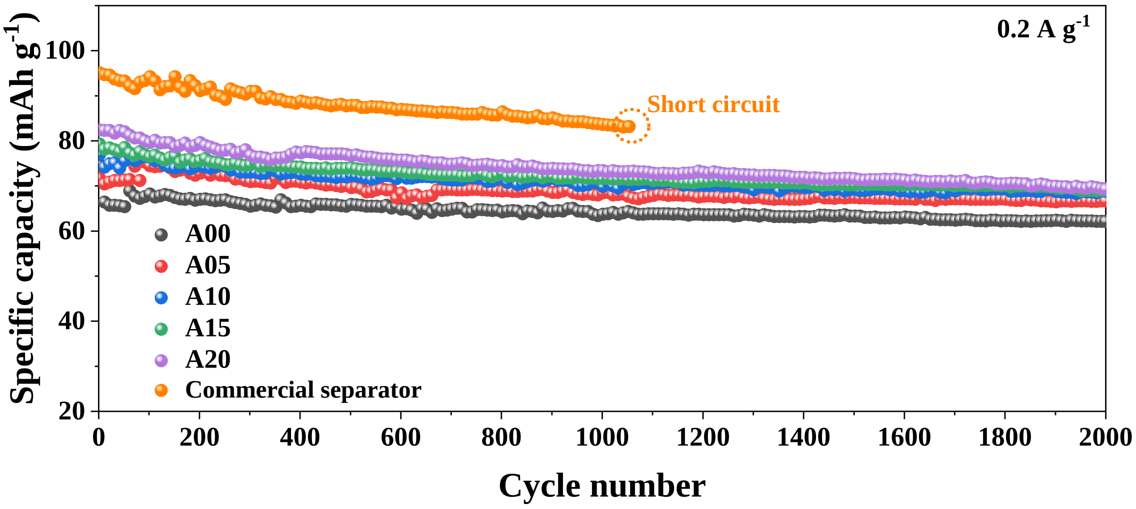

Fig. S17. Comparation of long-term cycling performance of groups with mortar separators and commercial separator measured at 0.2 A g^{-1}.

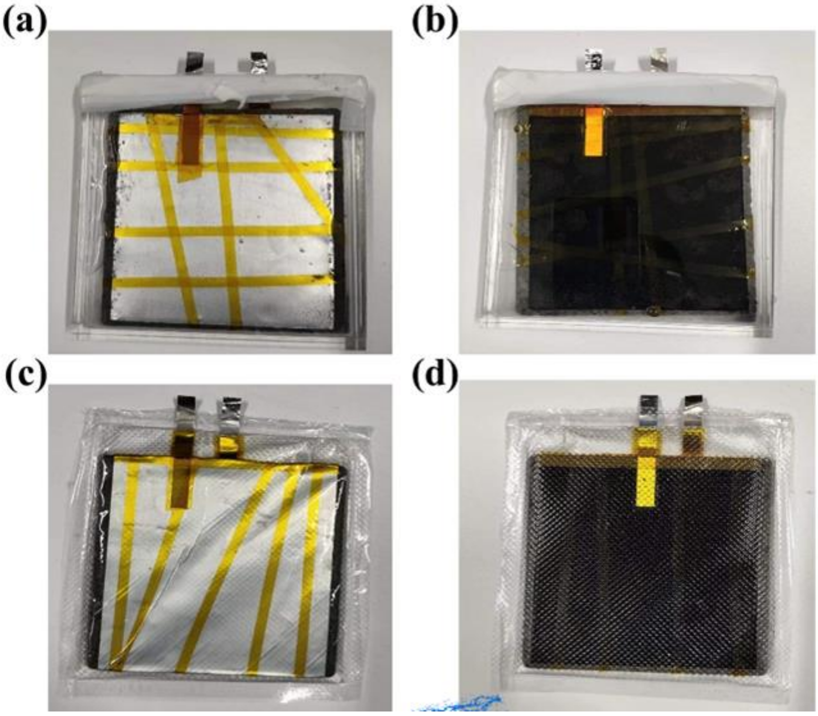

Fig. S18. Photographs of 100 cm^2 devices sealed in acrylic box from (a) anode view and (b) cathode view; sealed in transparent pouches from (c) anode view and (d) cathode view.

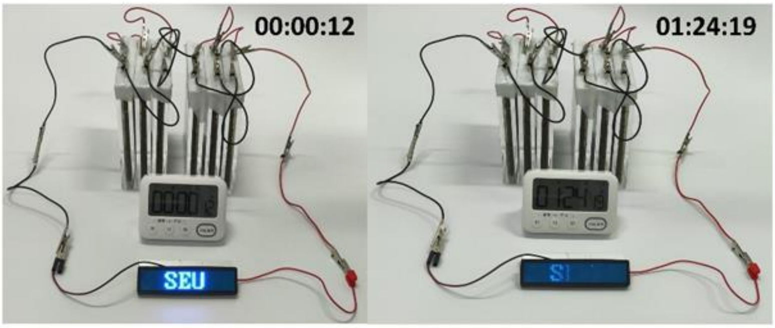

Fig. S19. Photographs of a set of 100 cm^2 devices illuminating a LED screen for a duration

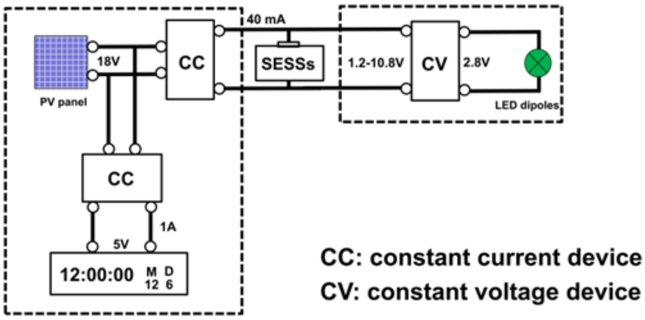

Fig. S20. Circuit schematic of a set of 100 cm^2 SESSs devices illuminating an LED screen for a duration

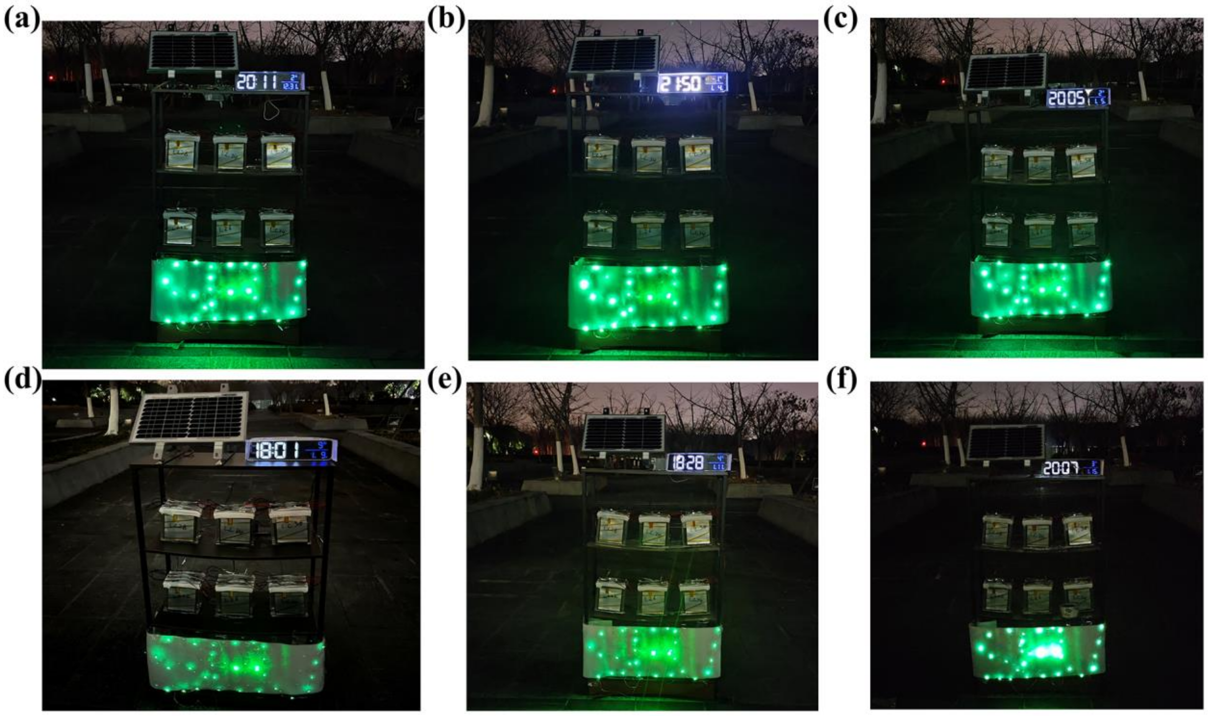

Fig. S21. Photographs of the concept demonstration of our device: outdoor experiments at Southeast University, Nanjing, China, from December 2023 to January 2024. (a)December 31, 2023, (b)January 4, 2024, (c) January 5, 2024, (d) January 9, 2024, (e) January 11, 2024, and (f) January 15, 2024.

3 Supplementary tables

Table S1 Mix proportions of mortar (in g)

Group	Cement	Water	Sand	AP	SDS
A00	100	70	100	-	-
A05	100	70	100	0.05	0.015
A10	100	70	100	0.10	0.015
A15	100	70	100	0.15	0.015
A20	100	70	100	0.20	0.015

Table S2 Compositions of samples from different groups (in mass ratio, wt.%)

Groups	$Ca(OH)_2$	$CaCO_3$	$CaSO_4 \cdot 2H_2O$	$Zn_4SO_4(OH)_6 \cdot 5H_2O$	$ZnSO_4 \cdot H_2O$	Others
A00-0d	14.0	3.1	-	-	-	82.9
A10-0d	13.7	3.4	-	-	-	82.9
A20-0d	13.3	3.6	-	-	-	83.1
A00-28d-N	17.2	9.1	-	-	-	73.7
A10-28d-N	10.8	21.3	-	-	-	67.9
A20-28d-N	7.1	32.3	-	-	-	60.6
A00-28d-$	16.0	3.5	-	-	-	80.5
A10-28d-$	-	3.2	27.4	20.9	6.5	42.0
A20-28d-$	-	2.9	21.4	19.9	18.3	37.4

Table S3 Summary of SESS from various literature sources compared in the article

Cathode	Anode	Separator	Electrolyte	Ref.
rGO/Fe_2O_3@foam Ni (Hydrothermal)	rGO@foam Ni (Hydrothermal)	Cement pastes with 10 wt% PAA and 4 wt% $K_3Fe(CN)_6$	10wt% KOH inner dosage	[1]
NiO_x@carbon mesh in cement paste with 0.9 vol% carbon fiber (Electrodeposition)	Fe@carbon mesh in cement paste with 0.9 vol% carbon fiber (Electrodeposition)	Mortar with 36 wt% ion exchange resin	10wt% KOH inner dosage	[2]
rGO@foam Ni (Commercial)	rGO@foam Ni (Commercial)	Cement pastes with 15 wt% hemp fiber	Soaking in 2M KOH	[3]
PEDOT on fired bricks (chemical deposition)	PEDOT on fired bricks (chemical deposition)	PVA gel	1M H_2SO_4	[4]
AC@SSM (Commercial)	Znic foil (Commercial)	Aerated mortar with 0.1 wt% AP dosage	Vacuum impregnated with 2M $ZnSO_4$	This work

Table S4 Properties of the SESS from various literature sources compared in the article

Energy density (Wh kg^{-1})/F_{ED}	Areal energy density (Wh m^{-2}) /FA_{ED}	Cycle number (capacity decreased by 90%)/F_{CN}	Reciprocal of cost (Wh/1000 ¥) /F_{RoC}	Compressive strength (MPa) /F_{CS}	Ref
19.8/3.9	489.1/5.0	6/1.0	178.4/4.2	18.0/5.0	[1]
20.3/3.9	51.9/3.4	1000/3.8	17.8/2.4	12.0/2.2	[2]
0.8/1.0	1.8/1.0	5000/4.6	3.1/1.0	13.2/2.9	[3]
8.7/3.1	121.0/4.0	10000/5.0	276.7/4.5	10.0/1.0	[4]
67.8/5.0	195.3/4.3	1200/3.9	533.6/5.0	15.4/3.9	This work

4 Supplementary discussion

4.1 Quantitative analysis of the reaction in $ZnSO_4$ soaking process

The quantitative analysis of the reaction products was conducted based on the results obtained from simultaneous thermal analyzer (STA). The presence of unreacted aerated mortar with $ZnSO_4$ allows the detection and quantification of CH and calcite, which exhibits distinct decomposition temperature regions labelled as Ⅰ and Ⅱ, respectively (**Fig. S6**), similar to previous findings [5]. Based on the in-situ XRD results shown in **Fig. S7**, the phase decomposition of aerated mortar soaked in $ZnSO_4$ at elevated temperatures [6] is summarized using Formula (S1)-(S7) along with the corresponding calculated values of each component in **Table S2**.

It should be noted that the amount of CH is utilized to determine the degree of cement hydration [7]. During the hydration process in a curing room, crystalline CH undergoes carbonation to generate calcite. However, when crystalline CH contacts with $ZnSO_4$, it converts to ZSH and gypsum. According to the STA results, the amount of crystalline CH, carbonate combined CH and sulfate combined CH can be calculated (**Fig. S8**).

For the aerated mortar soaked for 28 days, the amount of carbonate combined CH is slightly higher than the initial state while the total hydration degree remains equivalent to the 'A00-28d-$' group. It indicates that some factors hind the hydration reaction even as the solution permeates into the specimen. It is speculated that a passivation layer forms on the surface of cement particles, consisting of gypsum and ZSH, in a high concentration sulfate environment. This hinders the further hydration of cement and underscores the long-term stability as observed in terms of compressive strength and ionic conductivity.

4.2 Morphology of the ASM

The SEM images reveal that the group without AP in its initial state only exhibits a limited number of spherical pores (**Fig. S9a**), whereas the group with AP displays a rough surface (**Fig. S9b**) attributed to the presence of aerated pores. Typical hydration products of cement comprise crystalline CH, globular calcium silicate hydrate (C-S-H) and needle-like ettringite **(Fig. S9c)**. Additionally, cubic calcite is exclusively formed during carbonation (**Fig. S9d-e**) [8, 9]. Furthermore,

with the increase of AP dosage, larger crystal of ZSH and gypsum crystals are observed, indicating limited hindrance to crystal growth caused by the pore walls (**Fig. S9f**).

4.3 Discussion for the pore structure evolution via CT

In order to quantify the changes in pore structure, the frequency and porosity of pores in five size regions (0.05-0.20 mm, 0.20-1.00 mm, 1.00-4.50 mm, and 4.50-20.0 mm) were statistically analyzed in **Fig. S10a** and **Fig. S10b**. The results indicate that in the early stage, the pores ranging 0.05 to 0.20 mm possesses the highest frequency but the lowest porosity due to the small size. In contrast, pores ranging from 4.50 to 2.0 mm has the lowest frequency but the highest porosity.

Upon reacting with $ZnSO_4$, the large pores in the 4.50-20.0 mm range are filled and separated by the reaction products, leading to the formation of smaller pores and an overall increase in pores ranging in 0.05-4.50 mm. Notably, when the AP dosage is 0.1 wt.%, the occupied porosity of pores in the 1.00-4.50 mm range exceeds that in the 4.50-20.0 mm range, resulting in the refinement of the pore size. This refinement greatly contributes to the improvement in mechanical properties.

4.4 Discussion for the pore structure evolution of A00 group via MIP

As shown in **Fig. 3e**, the 'A00-0d' group had a few macro pores, a large number of capillary pores and a limited presence of gel pores in its initial state. When the specimen was soaked in $ZnSO_4$ solution, hydration of cement was the main reaction occurred. Consequently, in 'A00-0d-$', the volume of macro pores slightly decreases, the volume and average size of capillary pores decrease significantly, while gel pores increase significantly. This change is a result of the formation of hydration product, although not as pronounced as observed in 'A00-0d-N' **(Fig. S11a)**.

Reference:

[1] Wang J, Zhan P M, Zhang D. Redox active cement-based electrolyte towards high-voltage asymmetric solid supercapacitor. *Cem Concr Compos* 2023; **138:** 104987-105001.
[2] Zhang E Q, Tang L P. Rechargeable Concrete Battery. *Buildings* 2021; **11:** 103-117.
[3] Fang C Q, Zhang D. Pore forming with hemp fiber for magnesium phosphate structural supercapacitor. *Mater Des* 2020; **186:** 108322-108331.
[4] Wang H M, Diao Y F, Lu Y *et al.* Energy storing bricks for stationary PEDOT supercapacitors. *Nat Commun* 2020; **11:** 3882-3890.
[5] Soin A V, Catalan L J J, Kinrade S D. A combined QXRD/TG method to quantify the phase composition of hydrated Portland cements. *Cem Concr Res* 2013; **48:** 17-24.
[6] Peng H E, Xiang-Dong G A O, Li-Bin W U *et al.* Porous ZnO Sheets Transformed from Zinc Sulfate Hydroxide Hydrate and Their Photoluminescence Performance. *Acta Phys-Chim Sin* 2013; **29:** 874-880.
[7] Bai R, Zhang J, Yan C *et al.* Calcium hydroxide content and hydration degree of cement in cementitious composites containing calcium silicate slag. *Chemosphere* 2021; **280:** 130918.
[8] Xie M, Zhong Y, Li Z *et al.* Study on alkylsilane-incorporated cement composites: Hydration mechanism and mechanical properties effects. *Cem Concr Compos* 2021; **122:** 104161.
[9] Zhao D, Williams J M, Li Z *et al.* Hydration of cement pastes with calcium carbonate polymorphs. *Cem Concr Res* 2023; **173:** 107270.
